# Ultra-large alignments using phylogeny-aware profiles

**DOI:** 10.1186/s13059-015-0688-z

**Published:** 2015-06-16

**Authors:** Nam-phuong D. Nguyen, Siavash Mirarab, Keerthana Kumar, Tandy Warnow

**Affiliations:** Carl R. Woese Institute for Genomic Biology, University of Illinois at Urbana-Champaign, 1206 West Gregory Drive, Urbana, 61801 Illinois USA; Department of Computer Science, University of Texas at Austin, 2505 Speedway, Austin, 78712 Texas USA; Department of Bioengineering, University of Illinois at Urbana-Champaign, 1270 Digital Computer Laboratory, Urbana, 61801 Illinois USA; Department of Computer Science, University of Illinois at Urbana-Champaign, 201 North Goodwin Avenue, Urbana, 61801 Illinois USA

## Abstract

Many biological questions, including the estimation of deep evolutionary histories and the detection of remote homology between protein sequences, rely upon multiple sequence alignments and phylogenetic trees of large datasets. However, accurate large-scale multiple sequence alignment is very difficult, especially when the dataset contains fragmentary sequences. We present UPP, a multiple sequence alignment method that uses a new machine learning technique, the ensemble of hidden Markov models, which we propose here. UPP produces highly accurate alignments for both nucleotide and amino acid sequences, even on ultra-large datasets or datasets containing fragmentary sequences. UPP is available at https://github.com/smirarab/sepp.

**Electronic supplementary material:**

The online version of this article (doi:10.1186/s13059-015-0688-z) contains supplementary material, which is available to authorized users.

## Background

Multiple sequence alignments (MSAs) of large datasets, containing several thousand to many tens of thousands of sequences, are used for estimating the gene family tree for multi-copy genes (e.g., the p450 or 16S genes), estimating viral evolution, detecting remote homology, predicting the contact map between proteins [1], and inferring deep evolution [2]; however, most current MSA methods have poor accuracy on large datasets, especially for high rates of evolution [3, 4].

The difficulty in accurately estimating large MSAs is a major limiting factor in phylogenetic analyses of datasets containing several hundred sequences or more. Phylogeny estimation methods that do not require a MSA (e.g., truly alignment-free methods [5–7] or almost alignment-free methods such as DACTAL [4]) can be used, but alignments are necessary for estimating branch lengths, dates at internal nodes, detecting selection, etc. Therefore, phylogeny estimation generally uses methods such as maximum likelihood (ML) on estimated MSAs. ML phylogeny estimation on datasets containing thousands [8] to tens of thousands [9] of sequences is now feasible, but the accuracy of ML trees depends on having accurate MSAs [10], and estimating highly accurate large-scale alignments is extremely challenging; indeed, some datasets with only 1000 sequences can be difficult to align with high accuracy [11, 12].

**Fig. 1:**
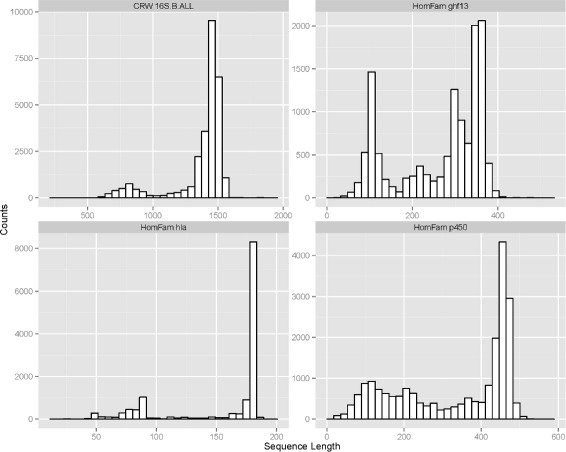
Histogram of sequence lengths for four of the biological datasets included in this study. These datasets show substantial sequence length heterogeneity and contain a mix of full-length and fragmentary sequences

Another challenge confronting MSA methods is the presence of fragmentary sequences in the input dataset (see Fig. 1 for examples of sequence length heterogeneity found in the biological datasets used in this study). This can result from a variety of causes, including the use of next-generation sequencing technologies, which can produce short reads that cannot be successfully assembled into full-length sequences.

We present a statistical MSA method that uses a new machine learning technique that we will introduce – the ensemble of hidden Markov models (HMMs) – to address these limitations. Each ensemble of HMMs is best seen as a collection of profile HMMs for representing a MSA, constructed in a phylogeny-aware manner; hence, we refer to this method as *UPP*, for *Ultra-large alignments using Phylogeny-aware Profiles*.

**Fig. 2:**
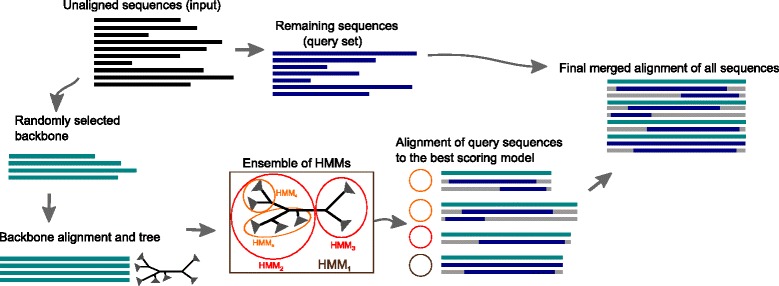
Overview of the UPP algorithm. The input is a set of aligned sequences. This sequence dataset is split into two parts: the backbone dataset and the set of query sequences. An alignment and tree are estimated for the backbone dataset, and an ensemble of HMMs is constructed based on the backbone alignment and tree. The query sequences are then aligned to each HMM, and the best scoring HMM for each sequence is used to add the query sequence to the backbone alignment. See text for more details

UPP uses the HMMER [13] suite of tools (see “Materials and methods”) to produce an alignment, and builds on ideas in SEPP [14]. The basic idea behind UPP is to estimate an accurate alignment for a subset of the sequences and align the remaining sequences to the alignment using profile HMMs [15]. UPP has four phases (see Fig. 2).

Phase 1 begins with unaligned sequences and selects a subset (called the “backbone dataset”) of the sequences; the remaining sequences are the “query sequences”. Phase 2 uses PASTA [16, 17] to compute a MSA and ML tree (which is unrooted) on the backbone sequences; these are called the “backbone alignment” and “backbone tree”, respectively. As PASTA is a global alignment method and is not designed for the alignment of fragmentary sequences, UPP preferentially selects the backbone sequences from those that are considered to be full length. To determine which sequences are “full length”, UPP only includes backbone sequences within 25 % of the length of the typical sequence for the given locus. If the typical length of the locus is not known, we use the median length of the input sequences as an estimate of the average length for thelocus.

This part of UPP’s algorithmic design is similar to alignment methods that are based on seed alignments (e.g., the technique used in Infernal [18]), but there is a basic difference between using seed alignments and these backbone alignments estimated by PASTA. Seed alignments are pre-computed alignments that are typically highly curated, and may be based on experimentally verified structural features of the molecule. UPP does not need to have such seed alignments, and instead is an entirely *de novo* alignment method.

Phase 3 creates a collection of HMMs (called the ensemble of HMMs) using the backbone alignment and backbone tree. The process begins by including the HMM computed on the entire backbone alignment. Next, the backbone tree is decomposed by removing a centroid edge (i.e., an edge that splits the tree into two subtrees of approximately equal size). For each of these two unrooted subtrees, we use hmmbuild (a command within HMMER) to compute an HMM on the backbone alignment restricted to the sequences in the leaf set of the subtree, and then add the resulting HMM to the ensemble. We repeat this decomposition process until each subtree contains at most ten sequences. Thus, this process results in an ensemble of HMMs, each computed on an alignment induced by the backbone alignment on one of the subtrees. Note also that while the subtrees are local regions within the backbone tree, they may not be clades within the tree (e.g., in Fig. 2, HMM _5_ is not based upona clade).

By default, hmmbuild combines nearby sites with more than 50 % gaps into a single match state, making it impossible to form a one-to-one mapping between the match states and the gappy sites in the original subset alignment. We modify the hmmbuild options to create a match state for each site that has at least one non-gap character, thus making it trivial to map the match states back to the original sites in the subset alignment.

Phase 4 inserts the remaining query sequences into the backbone alignment, as follows. The fit of each query sequence to each HMM is assessed using hmmsearch (a command within HMMER); this returns a bit score, which is a measure of the quality of the match between the query sequence and the HMM. The subset HMM with the best bit score is selected, and the sequence is inserted into the subset alignment using hmmalign (a command within HMMER). We treat each site within an alignment as a statement of positional homology, so that all letters within the site are considered to be positionally homologous [19]. Since positional homology is an equivalence relation (i.e., a binary relation that is reflexive, symmetric, and transitive), this process uses transitivity to define how the query sequence should be added into the backbone alignment; similar uses of transitivity have been used in other MSA methods [17, 20]. When the sequence has a letter (nucleotide or amino acid) that is not aligned to any letter in the backbone alignment, the extended alignment will have an “insertion site”.

Once all the query sequences have been added into the backbone alignment, the transitivity defines the final output MSA. This approach will tend to have potentially many insertion sites; to save space, we combine adjacent insertion sites into a single column. These introduced columns therefore contain nucleotides or amino acids that are not homologous to each other, and so the columns are indicated as insertion sites and masked before running a phylogenetic analysis. We also do not consider the homologies within these columns when evaluating the accuracy of computed alignments.

As we will show, UPP provides very good accuracy on both phylogenetic and structural benchmarks, and is fast enough to produce highly accurate alignments for 10,000 sequences in under an hour, and for one million sequences in 12 days, using only 12 cores. Furthermore, UPP has excellent accuracy even when the sequence dataset contains a large number of highly fragmentary sequences. In comparison, most other MSA methods either cannot analyze datasets of this size due to computational limitations, or do not exhibit the same accuracy as UPP under the most challenging conditions (large datasets with fragmentary sequences).

## Results and discussion

We used a variety of simulated and biological datasets from prior publications to compare UPP to existing MSA methods (see “Materials and methods” for details). The simulated datasets include ROSE NT (a collection of 1000-sequence nucleotide datasets), Indelible 10K (a collection of 10,000-sequence nucleotide datasets), RNASim (a collection of datasets ranging from 10,000 to one million sequences), and ROSE AA (a collection of 5000-sequence simulated amino acid datasets). The biological datasets include CRW (the three largest datasets, 16S.3, 16S.T, and 16S.B.ALL, from the Comparative Ribosomal Website [21] with up to 27,643 sequences), 10 AA (ten amino acid datasets with curated MSAs with up to 807 sequences), and HomFam (19 large HomFam datasets [22], with up to 93,681 sequences). For some of these datasets, we generated fragmented versions, making 12.5 %, 25 %, and 50 % of the sequences fragmentary, to evaluate the robustness to fragmentary data. The simulated datasets have true alignments and trees available from prior publications. The biological datasets have reference alignments based on structural features, and the CRW and 10 AA datasets also have reference trees computed using the reference alignments, which are also available from the prior publications. The reference alignments for the HomFam datasets are too small (5–20 sequences, median 7) and trees computed on these reference alignments were too poorly supported to be useful for evaluation purposes.

We computed ML trees on the estimated alignments, and report tree error using the false negative (FN) rate (also known as the missing branch rate), and the *Δ*FN rate, which is the difference between the FN rates of trees computed on estimated and true or reference alignments. We report alignment sum-of-pairs (SP) error, which is the average of the sum-of-pairs false negative (SPFN) and false positive (SPFP) rates [19]. We also report the total column score (TC), which is the percentage of aligned columns (i.e., columns with at least one homology) in the true or reference alignment that appear in the estimated MSA.

### UPP algorithm design

We explored modifications of the UPP design in which we varied the backbone size, used a single HMM instead of an ensemble, built ensembles based on clades within the backbone tree, built ensembles based on disjoint subsets of ten sequences each, used different MSA methods to compute the backbone alignment, used MAFFT instead of hmmalign to add sequences to the backbone alignment, and ran hmmbuild using different options to compute HMMs on each subset alignment. These preliminary studies revealed the following trends:

(1) Using small backbones (100 sequences) rather than large backbones (1000 sequences) typically produced higher alignment SP-error rates and tree error rates for both the ensemble of HMMs approach and the single HMM approach (Additional file 1: Sect. S2.1). Using smaller backbones reduced the running time for the ensemble of HMMs approach and had negligible impact on the running time for the single HMM approach (Additional file 1: Sect. S2.1).

(2) Using an ensemble of HMMs rather than a single HMM with 1000-sequence backbones had varying impact. As shown in Table 1, the impact on alignment SP-error ranged from neutral (changes of at most 0.3 % for alignment SP-score or tree error) to beneficial; for example, the alignment SP-error for the HomFam datasets using an ensemble of HMMs was 23.0 % whereas using a single HMM produced an alignment SP-error of 25.4 % (Table 1). The impact on TC score also varied: TC scores were better when single HMMs were used for the Indelible simulated datasets, and were otherwise better when ensembles were used (Table 1). The differences in TC score were generally small (e.g., the average difference was less than 0.5 %). For the HomFam datasets, using an ensemble of HMMs gave a TC score of 46.6 % while a single HMM had a TC score of 44.5 % (a difference of 2.1 %). For the Indelible 10000M4 datasets using a single HMM, the TC score was 30.5 %, and using an ensemble of HMMs the score was 27.4 % (a difference of 3.1 %).

**Table 1:** Comparison of two UPP variants on representative full-length datasets with respect to alignment SP-error, tree error, and TC scores

Model condition	Method	Alignment SP-error	*Δ*FN	TC score
10 AA	UPP (Default)	24.2	3.4	11.4
10 AA	UPP (Default, No Decomp)	24.5	5.2	11.0
ROSE AA	UPP (Default)	2.9	1.8	2.6
ROSE AA	UPP (Default, No Decomp)	2.8	1.4	2.5
CRW	UPP (Default)	12.5	7.8	1.4
CRW	UPP (Default, No Decomp)	13.3	16.5	0.9
HomFam (19)	UPP (Default)	23.0	NA	46.6
HomFam (19)	UPP (Default, No Decomp)	25.4	NA	44.5
Indelible 10000M2	UPP (Default)	3.5	0.6	1.2
Indelible 10000M2	UPP (Default, No Decomp)	3.3	0.5	1.4
Indelible 10000M3	UPP (Default)	1.3	0.2	4.6
Indelible 10000M3	UPP (Default, No Decomp)	1.3	0.1	4.8
Indelible 10000M4	UPP (Default)	0.3	<0.0	27.4
Indelible 10000M4	UPP (Default, No Decomp)	0.5	<0.0	30.5
RNASim 10K	UPP (Default)	9.5	0.8	0.5
RNASim 10K	UPP (Default, No Decomp)	11.2	3.0	0.3

All criteria (errors and scores) given as percentages. See text for explanation of names of methods and computational platforms used. The default setting for UPP is denoted UPP (Default); it uses a backbone of size 1000 and uses PASTA to compute the backbone alignment and the ensemble of HMMs technique. In the “No Decomp” versions of these two methods, the ensemble of HMMs is replaced with a single HMM. ML trees are estimated using RAxML (on the 10 AA datasets) or FastTree (all other datasets) except for HomFam, where we do not estimate ML trees as there are no reference trees for the HomFam datasets. *NA* Not applicable

Finally, using an ensemble of HMMs instead of a single HMM generally reduced tree error (Table 1). For example, results for the CRW datasets show that an ensemble of HMMs had an average tree error of 7.8 %, but using a single HMM had an average tree error of 16.5 % (i.e., more than double the tree error). Substantial reductions in tree error were also observed for the RNASim 10K datasets. In a few cases (i.e., the ROSE AA and Indelible datasets), using a single HMM improved tree error, but the differences were very small (Table 1). The impact of using an ensemble of HMMs instead of a single HMM was lessened for 100-sequence backbones, and in some cases even led to small improvements (Additional file 1: Sect. S2.1 and Additional file 1: Table S2.1). However, the best results were still obtained using the 1000-sequence backbones with the ensemble of HMMs.

(3) Using ensembles of HMMs computed for clades within the backbone tree produced alignments and trees that were generally as accurate (according to the SP-error and tree error rates) and had variable impact on TC scores (generally reducing scores but in some cases improving them) as those produced using ensembles based on the centroid-edge decompositions (Additional file 1: Sect. S2.6 and Additional file 1: Table S2.1). However, UPP using clade-based ensembles took more time (Additional file 1: Sect. S2.6).

(4) Using ensembles of HMMs based on disjoint subsets (each with at most ten sequences) had variable impact. For many datasets (e.g., the ROSE AA, RNASim, CRW, and HomFam datasets), the impact of using disjoint subsets was very small, and in some cases even slightly favorable (Additional file 1: Sect. S2.1 and Additional file 1: Table S2.1). However, for some other datasets, using disjoint subsets greatly reduced accuracy.

For example, for the Indelible 10000M2 datasets, default UPP had an alignment SP-error of 3.5 %, TC score 1.2 %, and *Δ*FN error of 0.6 %, but using disjoint subsets had SP-error of 28.2 %, TC score 0.3 %, and *Δ*FN tree error of 19.9 % (Additional 1: Table S1). Thus, although using disjoint ensembles of HMMs reduced the running time (Additional 1: Sect. S2.1), the default ensemble of HMMs was a more reliable technique than using ensembles based on disjoint subsets.

(5) The technique used to estimate the backbone alignment had a large impact on the final alignment and tree (Additional 1: Sect. S2.3), so that the final alignment SP-error very closely matched the initial backbone alignment SP-error (Additional 1: Sect. S2.4). Hence, the best alignment methods are needed to produce the backbone alignment.

(6) Using MAFFT to add sequences to the backbone alignment instead of UPP’s default technique (hmmalign, a command within HMMER) reduced accuracy (Additional 1: Sect. S2.5).

(7) Using different hmmbuild options (such as turning off the entropy-weighting flag) did not improve accuracy (Additional 1: Sect. S2.7).

Overall, the most reliable results were obtained using large backbones (1000 sequences), using an ensemble of HMMs, computing the backbone using PASTA, and using hmmalign to add sequences into the backbone alignment. These settings were used for the default version of UPP. However, for running-time purposes (so that ultra-large datasets can be analyzed quickly), we explore UPP (Fast), a variant of UPP that uses backbones of 100 sequences but otherwise uses all the default settings (i.e., it restricts the backbone to full-length sequences, it uses an ensemble of HMMs, it uses PASTA to align subsets, etc.).

### Comparison to other MSA methods for full-length sequences

We used Clustal-Omega [22], MAFFT [23], Muscle [24], PASTA [16, 17], and UPP to compute MSAs.

We rank methods by tiers, where the first tier contains the method that had the best performance as well as any other method that was within 1 % of the best result for the dataset. Similarly, the second tier contains the method not in the first tier that had the best performance, and all methods within 1 % of that method (and so forth for the remaining tiers). The method that had the best performance overall within a collection is also identified. We describe the general performance of each method on the full-length datasets (Table 2) and fragmentary datasets (Table 3). For the fragmentary results, we take the average performance of each method over the entire range of fragmented datasets.

**Table 2:** Average alignment SP-error, tree error, and TC score across most full-length datasets

Method	ROSE	RNASim	Indelible	ROSE	CRW	10 AA	HomFam	HomFam
	NT	10K	10K	AA			(17)	(2)
Average alignment SP-error								
UPP	7.8 (1)	9.5 (1)	1.7 (2)	2.9 (1)	12.5 (1)	24.2 (1)	23.3 (1)	20.8 (2)
PASTA	7.8 (1)	15.0 (2)	0.4 (1)	3.1 (1)	12.8 (1)	24.0 (1)	22.5 (1)	17.3 (1)
MAFFT	20.6 (2)	25.5 (3)	41.4 (3)	4.9 (2)	28.3 (2)	23.5 (1)	25.3 (2)	20.7 (2)
Muscle	20.6 (2)	64.7 (5)	62.4 (4)	5.5 (3)	30.7 (3)	30.2 (2)	48.1 (4)	X
Clustal	49.2 (3)	35.3 (4)	X	6.5 (4)	43.3 (4)	24.3 (1)	27.7 (3)	29.4 (3)
Average *Δ*FN error								
UPP	1.3 (1)	0.8 (1)	0.3 (1)	1.8 (1)	7.8 (2)	3.4 (2)	NA	NA
PASTA	1.3 (1)	0.4 (1)	<0.1 (1)	1.3 (1)	5.1 (1)	3.3 (1)	NA	NA
MAFFT	5.8 (2)	3.5 (2)	24.8 (3)	4.5 (3)	10.1 (3)	2.3 (1)	NA	NA
Muscle	8.4 (3)	7.3 (3)	32.5 (4)	3.1 (2)	5.5 (1)	12.6 (3)	NA	NA
Clustal	24.3 (4)	10.4 (4)	X	4.2 (3)	34.1 (4)	3.5 (2)	NA	NA
Average TC score								
UPP	37.8 (1)	0.5 (2)	11.0 (3)	2.6 (2)	1.4 (1)	11.4 (1)	47.3 (1)	40.3 (3)
PASTA	37.8 (1)	2.3 (1)	48.0 (1)	5.4 (1)	2.3 (1)	12.1 (1)	46.1 (2)	50.0 (1)
MAFFT	31.4 (2)	0.4 (2)	7.8 (4)	0.6 (3)	0.7 (2)	12.1 (1)	45.5 (2)	46.9 (2)
Muscle	9.8 (3)	<0.0 (2)	18.3 (2)	2.7 (2)	0.7 (2)	10.5 (2)	27.7 (4)	X
Clustal	5.7 (4)	0.2 (2)	X	3.1 (2)	0.1 (2)	11.8 (1)	38.6 (3)	31.0 (4)

We report the average alignment SP-error (the average of SPFN and SPFP errors) (top), average *Δ*FN error (middle), and average TC score (bottom), for the collection of full-length datasets. All scores represent percentages and so are out of 100. Results marked with an X indicate that the method failed to terminate within the time limit (24 hours on a 12-core machine). Muscle failed to align two of the HomFam datasets; we report separate average results on the 17 HomFam datasets for all methods and the two HomFam datasets for all but Muscle. We did not test tree error on the HomFam datasets (therefore, the *Δ*FN error is indicated by “NA”). The tier ranking for each method is shown parenthetically

**Table 3:** Average alignment SP-error and tree error across fragmentary datasets

Method	ROSE NT	RNASim 10K	Indelible 10K	CRW
				(16S.3 and 16S.T)
Average alignment SP-error				
UPP	8.3 (1)	11.8 (1)	2.7 (1)	16.1 (1)
PASTA	25.2 (2)	47.7 (4)	8.8 (2)	23.3 (2)
MAFFT	32.5 (3)	25.5 (2)	51.3 (3)	24.5 (3)
Muscle	35.3 (4)	82.2 (5)	77.6 (4)	70.6 (5)
Clustal	62.0 (5)	35.0 (3)	X	46.7 (4)
Average *Δ*FN error				
UPP	1.9 (1)	3.1 (1)	2.5 (1)	7.4 (2)
PASTA	25.2 (3)	21.9 (3)	9.0 (2)	8.2 (2)
MAFFT	18.0 (2)	6.2 (2)	35.6 (3)	2.5 (1)
Muscle	27.5 (4)	43.6 (5)	45.2 (4)	30.1 (3)
Clustal	47.8 (5)	26.3 (4)	X	37.4 (4)

We report the average alignment error (top) and average *Δ*FN error (bottom) on the collection of fragmentary datasets. Clustal-Omega failed to align any of the Indelible 10000M2 fragmentary datasets and thus we mark the results with an X. The tier ranking for each method is shown in parentheses

The majority of experiments were run on the homogeneous Lonestar cluster at the Texas Advanced Computing Center (TACC). Because of limitations imposed by Lonestar, these analyses are limited to 24 hours, using 12 cores with 24 GB of memory; methods that failed to complete within 24 hours or terminated with an insufficient memory error message were marked as failures. For experiments on the million-sequence RNASim dataset, we ran the methods on a dedicated machine with 256 GB of main memory and 12 cores until an alignment was generated or the method failed. We also performed a limited number of experiments on TACC with UPP’s internal checkpointing mechanism, to explore performance when time is not limited. All methods other than Muscle had parallel implementations and were able to take advantage of the 12 available cores.

On full-length datasets (Table 2) where nearly all methods were able to complete, PASTA was nearly always in the first tier with respect to alignment SP-error, tree error, and TC scores (the only exceptions being the RNASim 10K datasets where PASTA was in the second tier for alignment SP-error, and the HomFam (17) datasets where PASTA was in the second tier for TC score). UPP (Default) had the second best performance: it was in the first tier in terms of SP-error except for the Indelible 10K and HomFam (2) datasets, where it was in the second tier (with 1.2 % and 3.4 % higher error than the best method), it was in the first or second tier for tree error, and in the first through third tiers for TC score. MAFFT was in third place, being in the first through third tiers for alignment SP-error, first through third tiers for tree error, and first through fourth tiers for TC scores. Muscle and Clustal-Omega were behind MAFFT. Muscle was in the second through fifth tiers with respect to alignment SP-error, first through fourth tiers with respect to tree error, and second through fourth tiers with respect to TC score. Clustal-Omega was in the first through fourth tiers with respect to alignment SP-error, second through fourth tiers with respect to tree error, and first through fourth tiers with respect to TC scores. In general, the relative performance of Muscle and Clustal-Omega seemed to depend on the type of data, with Muscle doing better on the nucleotide datasets and Clustal-Omega doing better on the amino acid datasets.

Thus, for full-length sequences, whether with respect to alignment SP-error, tree error, or TC scores, on average PASTA came in first place, UPP in second, and MAFFT in third, while Muscle and Clustal-Omega were behind these methods.

### Comparison to other methods on datasets with fragmentary sequences

We next investigated performance for datasets with fragmentary sequences. As shown in Table 3, UPP was in the first tier of methods for all the fragmentary datasets with respect to alignment SP-error, and in the first tier of methods for three of the four collections (except for CRW) with respect to tree error, where it is in the second tier. PASTA was not in the first tier for any collection with respect to either criterion, and was instead in the second through fourth tiers for alignment SP-error and second and third tiers for tree error. MAFFT was in the second and third tiers for alignment SP-error, but did reasonably well for tree error: in the first tier for CRW and otherwise in the second and third tiers. As before, Muscle and Clustal-Omega did less well than the other methods; they were in the third through fifth tiers. Clustal-Omega was unable to analyze at least one dataset. Note also that the absolute error generally increased, and that only UPP had reasonably low alignment SP-error and tree error across all these fragmentary datasets. Thus, the relative and absolute performance of methods changed between the full-length and fragmentary data.

**Fig. 3:**
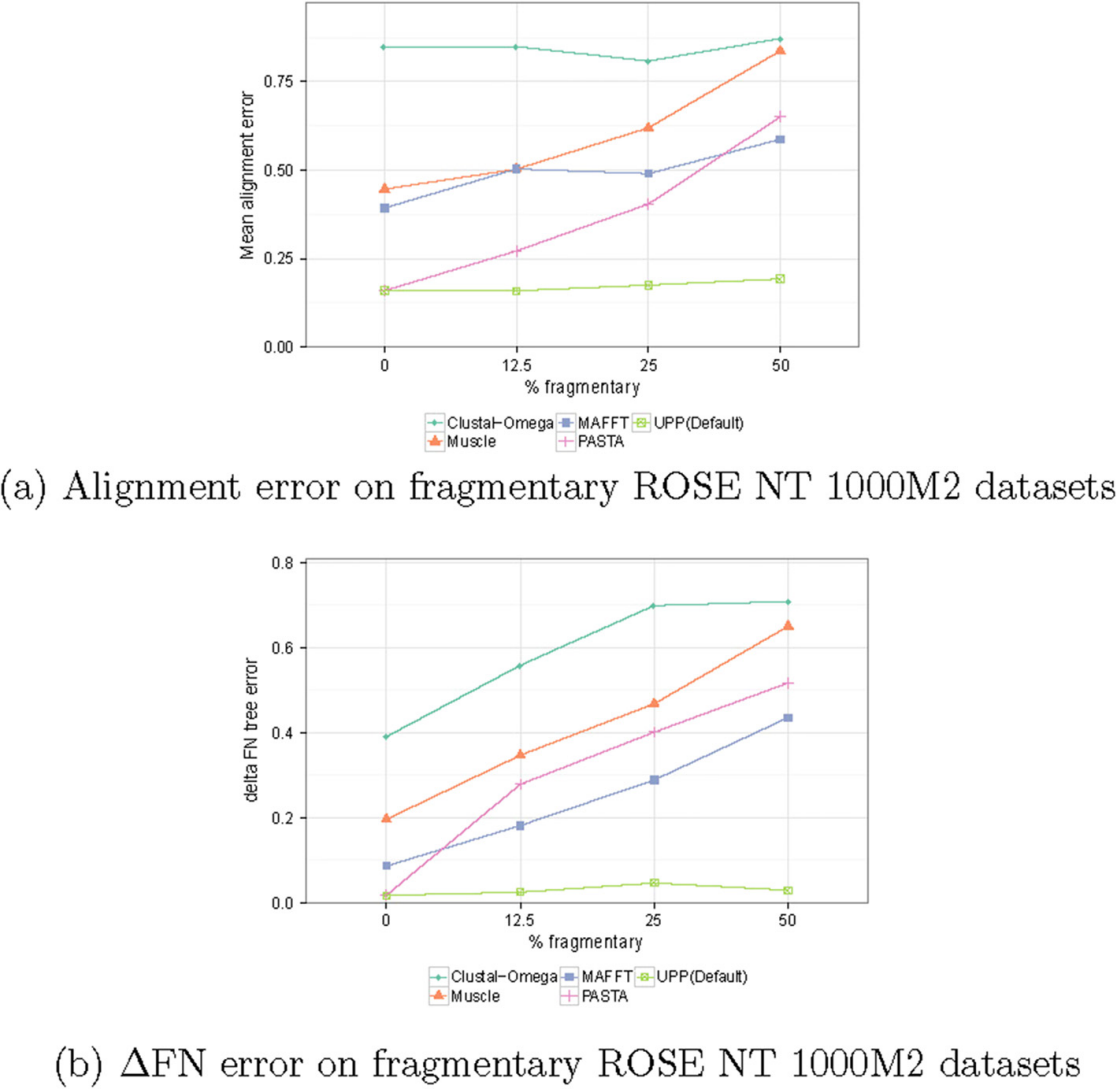
Impact of fragmentary sequences on alignment SP-error and tree error. We show average **a** alignment error and **b** *Δ*FN error rates for different methods for the ROSE NT 1000M2 datasets, but include results where a percentage of the sequences are made fragmentary, varying the percentage from 0 % to 50 %. Fragmentary sequences have average length 500 (i.e., roughly half the average sequence length for ROSE 1000M2)

Figure 3 shows the impact of fragmentation in detail. It has results for ROSE NT 1000M2 (a very challenging condition due to high rates of indels and substitutions), with varying levels of fragmentation.

UPP’s alignment SP-error increased only slightly with increases in fragmentation, even up to the highest degree of fragmentation (50 %). All other methods exhibited greater increases in alignment SP-error or tree error than UPP, as the amount of fragmentation increased.

**Fig. 4:**
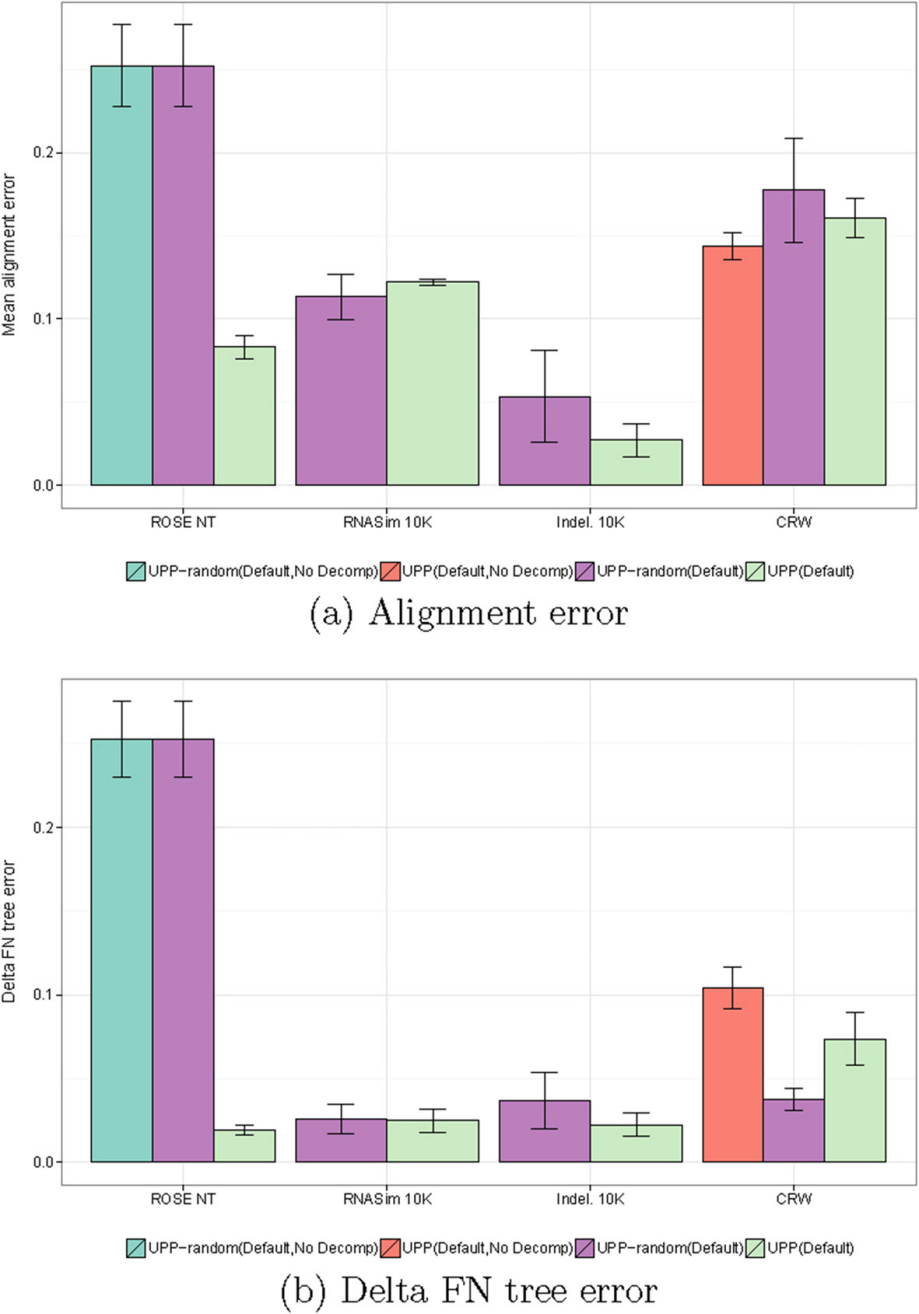
Comparison of UPP variants on fragmentary datasets. We show average **a** alignment error and **b** *Δ*FN tree error for UPP (Default), UPP (Default, NoDecomp), UPP-random (Default), and UPP-random (Default, NoDecomp) for the fragmentary datasets. The backbone is not restricted to full-length sequences in UPP-random, and so it allows fragmentary sequences in the backbone set. UPP-random (Default, NoDecomp) failed to align at least one dataset from each of the RNASim 10K, Indelible 10K, and CRW model conditions. UPP (Default, NoDecomp) failed to align at least one dataset from each of the ROSE NT, RNASim 10K, and Indelible 10K model conditions. ML trees were estimated using FastTree under the general time reversible model

To understand better why UPP is robust to fragmentation, we explored UPP variants (called UPP-random) in which we did not constrain the backbone to be only full-length sequences. We also looked at whether using the ensemble of HMMs instead of a single HMM contributes to robustness to fragmentation. These comparisons (Fig. 4) revealed some interesting trends about the impact of these algorithm design parameters. First, the only UPP variants that were able to align all the datasets were the two that used the ensemble of HMMs; the variants that used a single HMM each failed to align several datasets because HMMER was not able to align some of the query sequences to the backbone alignment (Fig. 4).

Second, the comparison between UPP-random (Default) and UPP (Default)) favored UPP (Default), so that while there were negligible to small differences in some cases, UPP (Default) was dramatically more accurate than UPP-random (Default) for the ROSE NT datasets for both alignment SP-error and tree error (Fig. 4). Thus, restricting the backbone to full-length sequences is a very important contribution to robustness to fragmentary sequences.

However, restricting the backbone to full-length sequences and using only a single HMM produced much higher tree error than using an ensemble of HMMs (Fig. 4), showing that using an ensemble of HMMs also provides benefits. Thus, the two algorithmic techniques (restricting the backbone to full-length sequences and using an ensemble of HMMs) are both useful in improving robustness to fragmentary sequences, but they address different analytical challenges.

### Impact of taxon sampling

**Fig. 5:**
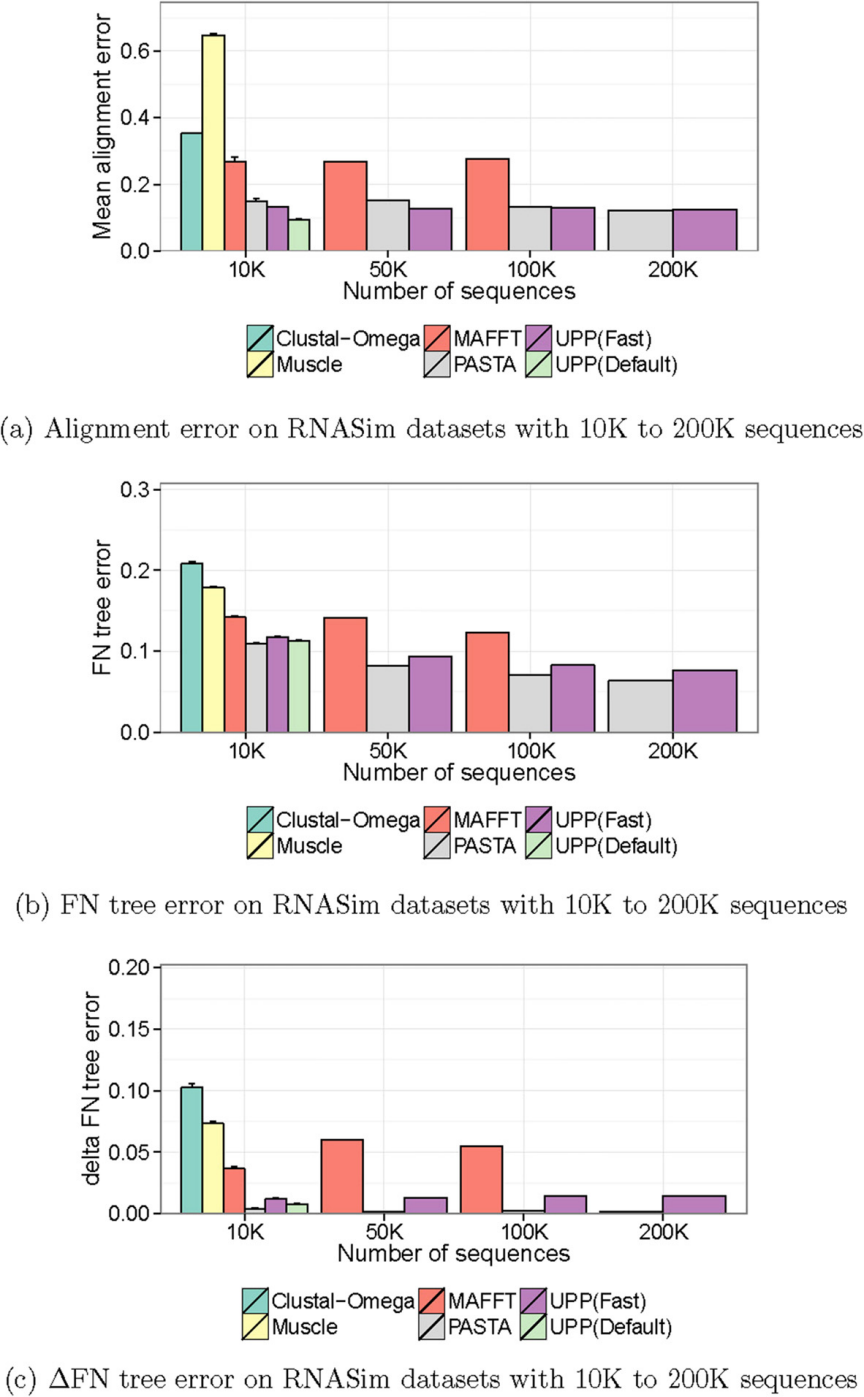
Comparison for the RNASim 200K dataset. We show **a** alignment SP-error, **b** FN tree error, and **c** *Δ*FN tree error rates for RNASim datasets with up to 200K sequences. Results not shown are due to methods failing to return an alignment within the 24-hour time period on TACC using 12 cores. ML trees were estimated using FastTree under the general time reversible model

We evaluated the ability of different methods to analyze very large datasets (up to one million sequences), using subsets of the million-sequence RNASim dataset; this comparison also reveals the impact of taxon sampling on the alignment methods. We examined performance for UPP (Fast), the fast version of UPP that differs from the default setting of UPP only in that it uses smaller backbones (100 sequences instead of 1000). Figure 5 shows results for 10,000 to 200,000 sequences, and compares UPP (Fast), UPP (Default), PASTA, MAFFT, Muscle, and Clustal-Omega, limiting analyses to 24 hours on a 12-core 24 Gb machine. While all methods shown were able to complete analyses for the 10K dataset, only UPP (Fast) and PASTA completed analyses for the 100K and 200K datasets.

As the number of sequences in the RNASim datasets increased, PASTA’s alignment SP-error dropped from 15.0 % at 50,000 sequences to 12.2 % at 200,000 sequences. UPP (Fast) had stable alignment SP-error across all the datasets, varying between 12.5 % and 13.3 %. The trees for both UPP and PASTA improved with increased taxon sampling, with PASTA trees approaching the accuracy of ML for the true alignment (0.1 % to 0.2 % *Δ*FN), and the UPP trees were close behind (1.2 % to 1.4 % *Δ*FN, Fig. 5c).

We then compared UPP (Fast) to PASTA for the full million-sequence RNASim dataset. We ran UPP (Fast) and PASTA on a dedicated machine with 12 cores and 256 GB of memory so that the analyses could exceed the 24 hour time limit in TACC. UPP (Fast) completed in 12 days, with alignment and tree errors similar to previous results (12.8 % alignment SP-error and 2.0 % *Δ*FN). PASTA completed in 15 days, and produced a much worse alignment but better tree errors (18.5 % alignment SP-error and 0.4 % *Δ*FN). Because we used a different machine with a different architecture, the running times for the million-sequence RNASim dataset cannot be directly compared to the running times for the other RNASim datasets, which were run on TACC.

### Computational issues

Table 4 compares wall-clock running times, using 12 cores, for those datasets where all methods were able to complete within the 24-hour limitation on Lonestar; thus, we show results for all datasets except for the RNASim datasets with 50K or more sequences. Note that all methods but Muscle had parallel implementations and were able to take advantage of the 12 available cores; the relative performance differences between methods could differ greatly on a single-core machine, depending on how well each method is able to take advantage of parallelism.

**Table 4:** Average wall-clock running time (hr) across most full-length datasets

Method	ROSE	RNASim	Indelible	ROSE	CRW	10 AA	HomFam	HomFam
	NT	10K	10K	AA			(17)	(2)
UPP	0.6	6.7	6.7	0.2	11.6	<0.1	1.3	0.5
PASTA	0.6	3.9	1.3	0.2	3.2	0.2	1.5	1.3
MAFFT	0.4	0.1	1.4	<0.1	0.4	0.1	<0.1	0.1
Muscle	0.5	0.8	1.2	<0.1	5.9	0.2	1.3	X
Clustal	0.4	4.8	X	0.1	2.8	<0.1	0.3	0.3

Average wall-clock running time for the full-length datasets for which most methods could complete; this includes everything other than the RNASim datasets with 50,000 or more sequences. UPP is run in default mode. Results marked with an X indicate that the method failed to terminate within the time limit (24 hours on a 12-core machine). All methods but Muscle had parallel implementations and were able to take advantage of the 12 cores. Muscle failed to align two of the HomFam datasets; we report separate average results for the 17 HomFam datasets for all methods and the two HomFam datasets for all but Muscle.

The differences in average running time for these datasets were sometimes small (e.g., all methods completed analyses in 0.4 to 0.6 hours wall-clock time for the ROSE NT datasets with 1000 sequences, and in less than 0.2 hours wall-clock time for the 10 AA datasets with under 1000 sequences). However, for the CRW datasets, which could be quite large (nearly 28K sequences), the differences in average running time were large: UPP (Default) used 11.6 hours, Muscle used 5.9 hours, PASTA used 3.2 hours, Clustal-Omega used 2.8 hours, and MAFFT used only 0.4 hours. Overall, for these datasets, MAFFT was generally the fastest (or nearly so), and UPP (Default) generally the slowest.

We compared the wall-clock running time for each stage of the UPP algorithm for UPP (Default) and UPP (Fast) for two large nucleotide datasets: the RNASim 10K dataset with 10,000 sequences and the CRW 16S.B.ALL dataset with 27,643 sequences (Table 5). Only two steps – computing the backbone alignment and tree and searching for the best HMM – used more than a few minutes, even for the largest dataset. Computing the backbone alignment and tree took under an hour for UPP (Default) and under 8 minutes for UPP (Fast). However, searching for the best HMM for the query sequences took the most time. For UPP (Default), which had ten times as many HMMs as UPP (Fast), this step took nearly 16 hours for 16S.B.ALL and 7 hours for the RNASim 10K dataset, while UPP (Fast) used under 1.8 hours for the 16S.B.ALL dataset and 0.8 hours for the RNASim 10K dataset. Thus, the vast majority of the time for large datasets is spent searching for the best HMM. For very small datasets, the difference in running time between UPP (Default) and UPP (Fast) is small, but for very large datasets the differences in running time are substantially increased – close to an order of magnitude in difference.

**Table 5:** Wall-clock running time (hr) for UPP (Fast) and UPP (Default) for the RNASim 10K and CRW 16S.B.ALL datasets

	RNASim 10K		CRW 16S.B.ALL	
Stage	UPP (Fast)	UPP (Default)	UPP (Fast)	UPP (Default)
Building backbone	0.12	0.42	0.13	0.52
Building HMMs	<0.01	0.02	<0.01	0.02
Searching for best HMM	0.83	6.53	1.81	15.45
Aligning sequences	0.02	0.03	0.05	0.15
Merge alignments	0.01	0.01	0.01	0.02
Total time	0.99	7.01	2.01	16.16

Wall-clock running time (hr) for each stage of UPP (Fast) and UPP (Default) for the RNASim 10K (10,000 sequences) and CRW 16S.B.ALL (27,643 sequences) datasets, two of the largest nucleotide datasets. The UPP alignments were computed on TACC’s Lonestar Cluster machine. The vast majority of the running time was spent searching for the best HMM for the query sequences.

**Fig. 6:**
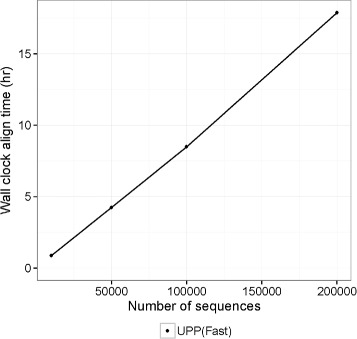
Running time for UPP (Fast) for the RNASim datasets. We show the running time to generate an alignment for UPP (Fast) for RNASim datasets with 10K, 50K, 100K, and 200K sequences. All analyses were run on TACC with 24 GB of memory and 12 CPUs

We then explored how UPP’s running time (measured using the wall-clock time) scaled with the size of the dataset by exploring subsets of the RNASim dataset with 10,000 to 200,000 sequences, using 12 cores. Running times for UPP (Fast) for the RNASim datasets showed a close to linear trend, so that UPP (Fast) completed for 10K sequences in 55 minutes, 50K sequences in 4.2 hours, 100K sequences in about 8.5 hours, and 200K sequences in about 17.8 hours (Fig. 6).

## Conclusions

Although the relative performance of MSA methods depended on the dataset, in most cases, UPP produced alignments with lower SP-error rates and higher TC scores than MAFFT, Muscle, and Clustal-Omega. ML trees computed with UPP alignments were also more accurate than ML trees for the other alignments. However, the comparison between UPP and PASTA is more interesting. Because UPP uses PASTA to compute its backbone alignment and tree, by design, UPP is identical to PASTA for fragment-free datasets containing at most 1000 sequences. The comparison between UPP and PASTA with respect to alignment accuracy is interesting: UPP alignments tend to have lower SP-error rates than PASTA alignments but also lower TC scores, indicating that these two criteria are not that well correlated. However, ML trees based on PASTA alignments (for fragment-free datasets) are typically more accurate than ML trees based on UPP alignments. For datasets with fragmentary sequences, UPP has nearly the same SP-error rates that it achieves with the full-length sequences, while PASTA’s SP-error rates increase substantially with fragmentation; consequently, UPP’s *Δ*FN tree error rates do not tend to increase that much with fragmentation although they do for PASTA. Thus, UPP is highly robust to fragmentary data whereas PASTA is not. Hence, while PASTA has an advantage over UPP for datasets without fragments, UPP presents advantages relative to PASTA for datasets with fragments.

To understand UPP’s performance, it is useful to consider the alignment strategy it uses. First, it computes a backbone alignment using PASTA for a relatively small (at most 1000-sequence) dataset; this allows it to begin with a highly accurate alignment. Then, instead of using a single profile HMM to represent its backbone alignment, UPP uses a collection of profile HMMs, each on a subset of the sequences. The subsets are obtained from local regions of the backbone tree, which is an ML tree estimated for the backbone sequences. Hence, the sequences in these subsets tend to be closely related. The induced subset alignments for these smaller localized regions are thus better suited for HMMs, especially when the full dataset displays overall substantial heterogeneity.

These observations help explain why using multiple HMMs, each for a region within the backbone tree, provides improved alignments compared to using a single HMM. However, UPP also restricts the backbone to the full-length sequences, and this algorithmic step is critical to improving robustness to fragmentary sequences. Hence, these aspects of UPP’s algorithmic design – restricting the backbone to full-length sequences and using an ensemble of HMMs instead of a single HMM –increase sensitivity to remote homology (especially for fragmentary sequences) and reduces alignment SP-error and tree error, but each targets a different aspect of algorithmic performance.

UPP exhibits great scalability with respect to running time (which scales in a nearly linear manner), parallelism, and alignment accuracy. For example, our study showed the alignment SP-error for the backbone alignment is quite close to the alignment SP-error for the alignment returned by UPP. Thus, UPP enables large datasets to be aligned nearly as accurately as smaller datasets.

Overall, UPP is a MSA method that can provide very high accuracy for sequence datasets that have been considered too difficult to align, including datasets with high rates of evolution, fragmentary sequences, or many thousands of sequences – even up to one million sequences. UPP performs well for both phylogenetic and structural benchmarks (see [25] for further discussion of these related but different tasks). Finally, UPP is parallelized (for shared memory) and has a checkpointing feature, but does not require supercomputers to achieve excellent accuracy for ultra-large datasets in reasonable time frames.

## Materials and methods

### Performance study

#### Data and software availability

The datasets used in this study are available at [26]. The GitHub site for UPP [27] provides open-source software and instructions on how to download, install, and run UPP.

#### Datasets

All the datasets used in our study have been used in previously published studies, and are available online through the respective publications. Because UPP is designed for ultra-large-scale MSA, we focus the analysis on benchmark datasets with many sequences. We used the following collections of simulated datasets:

- ROSE NT: a collection of 1000-sequence nucleotide datasets from [11] that were generated using ROSE [28]; see [11] for full details
- Indelible 10K: a collection of 10,000-sequence nucleotide datasets from [16] that were generated by Indelible [29]; see [16] for full details
- RNASim: a collection of datasets ranging from 10,000 sequences to one million sequences [17]
- ROSE AA: a collection of 5000-sequence simulated amino acid datasets from [9] that were generated using ROSE

We also used biological datasets with reference alignments that have been used in prior studies [12, 17, 22] to evaluate alignment methods for large datasets. We focus on datasets with 10,000 or more sequences, but also used ten large amino acid datasets (eight from the BAliBASE [30] collection and two others) with at least 320 sequences:

- CRW: The three largest datasets from the Comparative Ribosomal Website [21], each a set of 16S sequences. We include the 16S.3 dataset (6,323 sequences spanning three phylogenetic domains), the 16S.T dataset (7,350 sequences spanning three phylogenetic domains), and the 16S.B.ALL dataset (27,643 sequences spanning the bacteria domain). The CRW datasets have highly reliable, curated alignments inferred from secondary and tertiary structures and were previously studied in [3, 12]. The reference trees for these datasets used in these studies were derived from ML trees estimated using RAxML, with all branches with bootstrap support below 75 % collapsed.
- 10 AA: Ten amino acid datasets with curated MSAs (the eight largest BAliBASE datasets [30] and IGADBL_100 and coli_epi_100 from [31]); these range in size from 320 to 807 sequences, and were used in [17] to evaluate MSA methods. The reference trees for these datasets used in these studies were based on RAxML with all branches with bootstrap support below 75 % collapsed.
- HomFam: A collection of 19 of the largest HomFam datasets, which are amino acid sequence datasets ranging in size from 10,099 to 93,681 sequences with Homstrad [32] reference alignments for small subsets (5–20 sequences, median 7). These 19 datasets were used in [17, 22] to evaluate MSA methods for large amino acid datasets. The study in [22] also explored performance with smaller HomFam datasets, but these are not as relevant to this study. As noted in [17], there was a warning in the PFAM website regarding the HomFam rhv dataset studied in [22], stating that the alignment was “very weak”; for that reason, the rhv dataset was omitted from the study reported in [17] and from this one.

For some of the nucleotide datasets, we generated three fragmented versions, by making 12.5 %, 25 %, and 50 % of the sequences fragmentary. The lengths of the fragments were drawn from a normal distribution with a mean length of 500 bp and a standard deviation of 60 bp (the mean length is one-third of the average length of the CRW datasets and one-half the length of the Indelible and ROSE NT datasets). We generated fragmentary datasets by selecting a random subset of sequences and a random substring (of the given length) for each selectedsequence.

### Alignment and tree estimation software

Each dataset was aligned (when possible) using Clustal-Omega [22] version 1.2.0, MAFFT [23] version 6.956b, MUSCLE [24] version 3.8.31, and PASTA version 1.5.1 [16, 17]. MUSCLE was run with the -maxiters 2 option on datasets of 3000 sequences or greater. Due to a bug in earlier versions of MAFFT 6.956b, MAFFT-default was run using MAFFT version 7.143. We ran three different versions of MAFFT. MAFFT-L-INSI was run on datasets with 1000 or fewer sequences. For most datasets with more than 1000 sequences, we ran MAFFT-default (--auto); the exceptions were the RNASim 100K dataset, three replicates from the Indelible 10K 10000M3 dataset, and the CRW 16S.B.ALL dataset, where MAFFT-default failed to run and so we used MAFFT-PartTree. All MAFFT variants included the --ep 0.123 parameter.

Because the algorithmic design parameters for running PASTA on amino acid datasets has not been studied before, we examined different options for running PASTA on amino acid datasets and used those settings in our studies (see Additional 1: Sect. S3). PASTA was run for three iterations or a maximum of 24 hours, whichever came first. If PASTA did not terminate at the end of 24 hours, the alignment from the last successfully completed iteration was used. PASTA was run using a MAFFT-PartTree starting tree for all but the RNASim datasets. For the RNASim datasets, we used the ML tree estimated from the UPP (Fast, NoDecomp) alignment as the starting tree (MAFFT-PartTree was unable to run on the largest RNASim datasets). The remaining settings for PASTA were set using the --auto flag.

Commands for each method are given below:

- Clustal-Omega
  clustalo --threads = 12 -i<input_sequence >-o <output_alignment >
- MAFFT-L-INS-i
  mafft --ep 0.123 --thread 12 --localpair --maxiterate 1000 --quiet --anysymbol<input_sequence>><output_alignment>
- MAFFT-default
  mafft --thread 12 --ep 0.123 --auto --quiet --anysymbol<input_sequence>><output_alignment>
- MAFFT-PartTree
  mafft --thread 12 --ep 0.123 --parttree --retree 2 --partsize 1000 --quiet<input_sequence>><output_alignment>
- MAFFT-profile
  mafft [--localpair --maxiterate 1000] [--addfragment|--add]<query_file><backbone_alignment>><output_alignment>
- Muscle
  muscle [-maxiters 2] -in <input_sequence > -out <output_alignment >
- PASTA
  python run_pasta.py --num-cpus=12 -o<output_directory>
  -i<input_sequences>-t<starting_tree>--auto --datatype=<molecule_type>
- UPP
  python exhaustive_upp.py -a<backbone_alignment>-t<backbone_tree>-s<query_sequences>-d<output_directory>-o<output_name>-x 12 -A 10 -m<molecule_type>-c<default_config_file>
- UPP-disjoint
  python exhaustive_upp.py -S normal -a<backbone_alignment>
  -t<backbone_tree>-s<query_sequences>-d<output_directory>-o<<output_name>-x 12 -A 10 -m<molecule_type>-c<default_config_file>

### HMMER commands

HMMER 3.0 [13] was used internally within UPP for building the ensemble of HMMs (hmmbuild), for searching for the best HMM for a query sequence (hmmsearch), and for inserting the query sequence into the alignment (hmmalign):

- hmmbuild
  hmmbuild --symfrac 0.0 --informat afa --<molecule_type><output_profile><backbone_alignment>
- hmmsearch
  hmmsearch --noali -o<output_file>--cpu 1 -E 99999999
  --max<input_profile><query_file>
- hmmalign
  hmmalign --allcol --dna<output_profile><query_file><output_alignment>

### Maximum likelihood tree estimation

To compute ML trees for large datasets (with 1000 or more sequences), we used FastTree [9] version 2.1.5 SSE3, and we used RAxML [8] version 8.0.6 for smaller datasets. We used the general time reversible model for all the nucleotide datasets (simulated and biological) and JTT [33] for the simulated amino acid datasets (ROSE AA). For the 10 AA datasets (all biological), we used ProtEST [34] to select the model for each dataset, and then used that model within RAxML to perform the analysis. The commands used to run each method are givenbelow:

- FastTree AA
  FastTreeMP -nosupport<input _fasta>><output_tree>
- FastTree NT
  FastTreeMP -nosupport -nt -gtr<input_fasta>><output_tree>
- RAxML AA
  raxmlHPC -T 12 -m PROT<model_name>GAMMA -j -n<output_name>
  <starting_tree>-s <input_fasta > >-w<output_directory>-p 1

### Performance metrics

We compare estimated alignments and their ML trees to reference alignments and trees. We use FastSP [19] to compute SP-error (the average of SPFN and SPFP errors) and TC scores. The SPFN rate is the percentage of homologous pairs in the reference alignment that are not in the estimated alignment and the SPFP rate is the percentage of homologous pairs in the estimated alignment that are not present in the referencealignment.

We report tree error using the FN rate (also known as the missing branch rate), which is the percentage of internal edges in the reference tree that are missing in the estimated tree. We also report *Δ*FN, the difference between the FN rate of the estimated tree and the FN rate of the tree estimated on the true alignment, to evaluate the impact of alignment estimation on phylogenetic analysis. Most typically, *Δ*FN>0, indicating that the estimated tree has higher error than the ML tree for the true alignment, but it is possible for *Δ*FN<0, which happens when the estimated ML tree is more accurate than ML for the true alignment.

## Additional file

Additional file 1 **Supplementary materials discussed in the main paper.** This file is available at [35]. It contains information on early termination by alignment methods, comparisons of UPP variations, and an evaluation of parameter settings for PASTA, which is used for amino acid MSA.

## Abbreviations

*Δ*FN: delta false negative
FN: False negative
HMM: Hidden Markov model
MSA: Multiple sequence alignment
ML: Maximum likelihood
SP: sum of pairs
SPFN: Sum-of-pairs false negative
SPFP: Sum-of-pairs false positive
TACC: Texas Advanced Computing Center
TC: Total column
