# Supplementary material for: Ultra-large alignments using phylogeny-aware profiles
Source: Genome Biol. 2015 Jun 16;16(1):124. doi: 10.1186/s13059-015-0688-z (PMC4492008; doi:10.1186/s13059-015-0688-z)
Supplement: Supplementary file 1 — Supplementary materials discussed in the main paper. This file is available at [35]. It contains information on early termination by alignment methods, comparisons of UPP variations, and an evaluation of parameter settings for PASTA, which is used for amino acid MSA. [file 13059_2015_688_MOESM1_ESM.pdf]

# Supporting Online Materials

Nam-phuong Nguyen[1], Siavash Mirarab[2], Keerthana Kumar[2], and Tandy Warnow[1,3,4*]

[1]Carl R. Woese Institute for Genomic Biology, University of Illinois at Urbana-Champaign,
[2]Department of Computer Science, University of Texas at Austin,
[3]Department of Bioengineering, University of Illinois at Urbana-Champaign,
[4]Department of Computer Science, University of Illinois at Urbana-Champaign,
[*]To whom correspondence should be addressed; E-mail: warnow@illinois.edu.

June 8, 2015

# Contents

**S1 Early termination on large datasets** **5**

**S2 UPP pipeline exploration** **7**

S2.1 Comparison of a nested ensemble of HMMs, a disjoint ensemble of HMMs, and a single HMM . . . . . . . . . . 7

S2.2 Backbone size . . . . . . . . . . 13

S2.3 Backbone alignment method . . . . . . . . . . 13

S2.4 Backbone and final alignment SP-error . . . . . . . . . . 13

S2.5 Query sequence alignment method . . . . . . . . . . 15

S2.6 Comparison of clade-based versus centroid edge decomposition. . . . . . . . . . 15

S2.7 hmmbuild options . . . . . . . . . . 18

**S3 PASTA on the 10 AA datasets** **21**

**S4 Supplemental Tables** **25**

# List of Figures

S2.1 Alignment SP-error and tree error of UPP(Default), UPP(Default,Disjoint), and UPP(Default,NoDecomp) on the HomFam and CRW 16S datasets. . . . . . . . 9

S2.2 Alignment SP-error and tree error of UPP(Default), UPP(Default,Disjoint), and UPP(Default,NoDecomp) on the Indelible and ROSE AA datasets. . . . . . . . 10

S2.3 Alignment SP-error and ΔFN tree error for UPP using different methods backbone alignments on the RNASim 10K dataset. . . . . . . . . . . . . . . . . . . 14

S2.4 Comparison of initial backbone alignment SP-error and final UPP(Default) alignment SP-error. . . . . . . . . . . . . . . . . . . . . . . . . . . . . . 16

S2.5 Impact of backbone size and query sequence alignment method on alignment SP-error, tree error, and running time. . . . . . . . . . . . . . . . . . . . . . . . . 17

S2.6 Alignment SP-error and tree error of centroid-edge decomposition and clade-based decomposition on the CRW datasets. . . . . . . . . . . . . . . . . . . . . 18

S2.7 Alignment SP-error and tree error for different options of hmmbuild within UPP on the CRW datasets. . . . . . . . . . . . . . . . . . . . . . . . . . . . . . 20

S3.1 Alignment SP-error and tree error of PASTA variants on the 10 AA datasets with full reference alignments, using substitution models selected by ProtEST. . 22

S3.2 Alignment SP-error and tree error of different methods on the 10 AA datasets with full reference alignments, using substitution models selected by ProtEST. . 23

# List of Tables

S2.1 Comparison of UPP variants on representative full-length datasets. . . . . . . . 11

S2.2 UPP variants on the RNASim datasets. . . . . . . . . . . . . . . . . . . . . . . . 12

S4.1 FN tree error rates across the biological datasets. . . . . . . . . . . . . . . . . . . 25

S4.2 Alignment SP-error for the full-length biological datasets. . . . . . . . . . . . . . 26

S4.3 TC score for the full-length biological datasets. . . . . . . . . . . . . . . . . . . . 27

# Materials and Methods

## S1 Early termination on large datasets

Many alignment methods failed to complete analyses on the larger datasets, but reasons varied. Some failed due to insufficient memory, or due to a bug in the software, or were simply unable to produce an alignment within the 24 hour time limit (i.e., they might have been able to produce an alignment if given more time). This section documents each case.

**MAFFT-default.** MAFFT-default terminated early on the CRW 16S.B.ALL and three of the Indelible 10000M3 datasets. The error messages produced by MAFFT-default have the following template:

```
Cannot allocate <X> character vector.
```

where $X$ is a large number. MAFFT-default also failed to produce an alignment on the RNASim 100K dataset within the 24 hour time limit on TACC. According to MAFFT's output log, MAFFT was still running when the job was evicted.

**MAFFT-PartTree.** MAFFT-PartTree terminated with the following error message on the RNASim 200K dataset:

```
mafft: line 2028: 28963 Segmentation fault
"$prefix/splittbfast" $legacygapopt -Z
$algopt $splitopt $partorderopt $parttreeoutopt
$memopt $seqtype $model -f "-"$gop -Q
$spfactor -h $aof -p $partsize -s
$groupsize $treealg -i infile > pre 2>> "$progressfile"
```

**MUSCLE.** MUSCLE terminated early on the RNASim datasets with 50,000 or more sequences with the following error message:

```
*** OUT OF MEMORY ***
Memory allocated so far 23718.4 MB
No alignment generated
```

On the HomFam zf-CCHH and rvp datasets, MUSCLE terminated with the following error message: *Segmentation fault*.

**Clustal-Omega.** Clustal-Omega failed to terminate within 24 hours on the RNASim datasets with 50,000 or more sequences. The log file showed that Clustal-Omega was still running, so given enough time, it may be possible for Clustal-Omega to produce an alignment on the larger RNASim datasets.

On the Indelible 10000M2 dataset, Clustal-Omega terminated early with the following error message:

```
HHalignWrapper:hhalign_wrapper.c:945: problem in
alignment (profile sizes: 892 + 1540) (S1870 + S7661),
forcing Viterbi
        hh-error-code=3 (mac-ram=2048)
+-------------------------------+
| both sequences truncated right |
+-------------------------------+
i2 = 2 != 6699 = qa->L, j2 = 10788 != 10846 = ta->L
PrintAlignments:hhhitlist-C.h:199: qt_ali.Build failed
hhalign:hhalign.cpp:1216: Could not print alignments
```

```
HHalignWrapper:hhalign_wrapper.c:984: 2nd attempt
worked HHalignWrapper:hhalign_wrapper.c:945:
problem in alignment
(profile sizes: 833 + 2432) (S3589 + S1870), forcing Viterbi
        hh-error-code=3 (mac-ram=2048)
```

# S2 UPP pipeline exploration

We explored the impact of changes to the UPP pipeline on alignment SP-error and tree estimation error.

## S2.1 Comparison of a nested ensemble of HMMs, a disjoint ensemble of HMMs, and a single HMM

We compared UPP to two different techniques for representing the backbone alignment (Table S2.2 and Figs. S2.1-S2.2). The first technique used a single HMM to represent the backbone alignment (UPP with no decomposition); this is equivalent to using HMMER to align the query sequences. The second technique used disjoint subsets of at most 10 sequences generated by using a centroid decomposition to represent the backbone alignment.

We report the average alignment SP-error, $\Delta$FN error (the difference between the error of the ML tree estimated on the true alignment and the ML tree estimated on the estimated alignment), and wall clock running time (in hours). Methods run on the datasets containing 10K to 200K sequences were run on a dedicated node with 12 processors with 24Gb of memory. Methods run on the 1,000,000 sequence dataset were run on a dedicated machine with 12 processors and 256 Gb of memory. Thus, the running times of the methods run on the 10K to 200K datasets cannot be directly compared to the methods run on the 1M dataset. We mark the running times of the methods that were run on the 1M dataset with "*". The default setting for UPP is

denoted UPP(Default); it uses a backbone of size 1000, uses PASTA to compute the backbone alignment, and the ensemble of HMMs technique; UPP(Fast) is obtained by using backbones of size 100 and keeping all other settings constant. The "NoDecomp" versions of these two methods replace the ensemble of HMMs technique with a single HMM. The "Disjoint" versions of these methods use HMMs computed on disjoint subset alignments of at most ten sequences. UPP(Default,Clade) uses clade-based decompositions to generate the sequence subsets.

We begin with a discussion of the impact of using a single HMM instead of an ensemble of HMMs. On the RNASim datasets, using a single HMM instead of an ensemble of HMMs clearly increased alignment SP-error and tree error (Table S2.2). Using a single HMM instead of an ensemble did not change alignment SP-error on the CRW datasets but did increase tree error substantially, and using a single HMM instead of an ensemble also increased alignment SP-error on the HomFam datasets (Fig. S2.1). Finally, using a single HMM instead of an ensemble of HMMs had no impact in either alignment or tree error on the Indelible 10K or ROSE AA datasets (Fig. S2.2).

Using disjoint HMMs instead of an ensemble of HMMs increased alignment SP-error and tree error on the Indelible datasets with 10K sequences and higher evolutionary rates (10000M2 and 10000M3) and had no impact on the lowest evolutionary rate (10000M4) (Fig. S2.2). Using disjoint HMMs increased alignment SP-error but not tree error on the RNASim 10K datasets, and had no impact on the RNASim 50K datasets (Table S2.2). Using disjoint HMMs did not impact alignment SP-error or tree error on the ROSE AA datasets (Fig. S2.2). Using disjoint HMMs had no impact on the alignment SP-error or tree error for the CRW datasets, and slightly reduced average alignment SP-error on the HomFam datasets (Fig. S2.1).

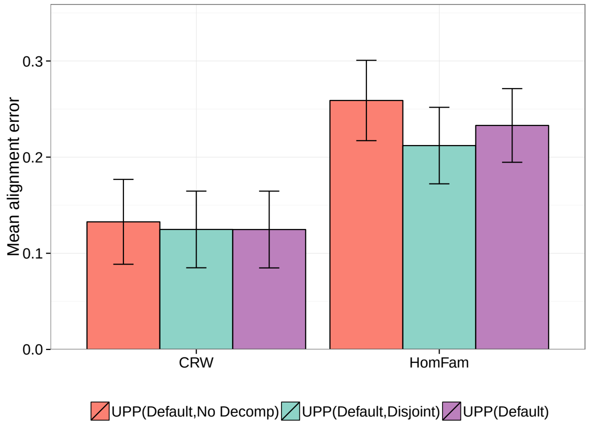


(a) Alignment SP-error

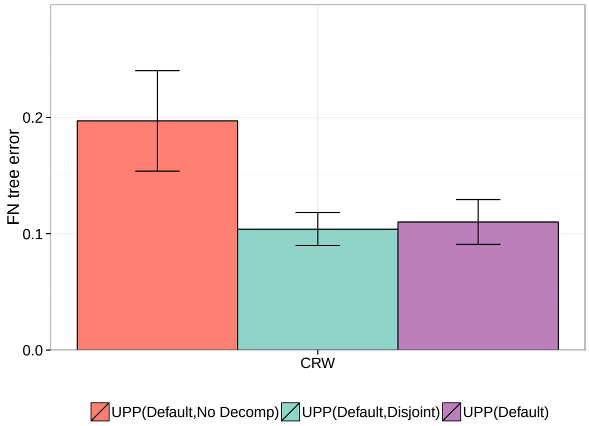


(b) Tree error

Figure S2.1: **Alignment SP-error and tree error for UPP(Default), UPP(Default,Disjoint), and UPP with no decomposition on the HomFam and CRW 16S datasets.** All methods used a backbone size of 1000. UPP(Default,NoDecomp) uses a single HMM to align the query sequences. UPP(Default,Disjoint) uses disjoint HMMs of at most 10 sequences. Note that we do not have reference trees for the HomFam datasets, and thus, do not report tree error on the HomFam datasets. ML trees were estimated using FastTree under GTR.

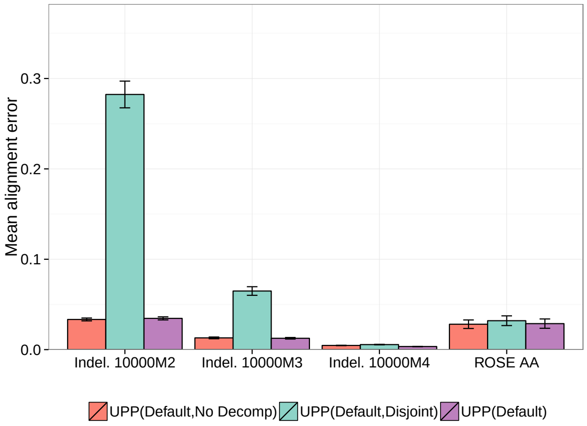


(a) Alignment SP-error

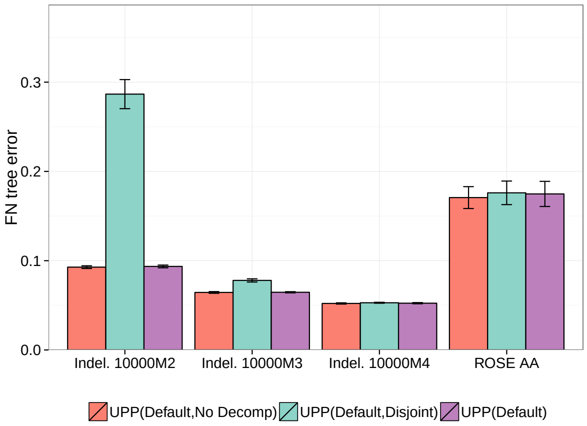


(b) Tree error

Figure S2.2: **Alignment SP-error and tree error for UPP(Default), UPP(Default,Disjoint), and UPP(Default,NoDecomp) on the Indelible and ROSE AA datasets.** All methods use a backbone size of 1000. UPP(Default,NoDecomp) uses a single HMM to align the query sequences. UPP(Default,Disjoint) uses disjoint HMMs of at most 10 sequences. ML trees are estimated using FastTree under GTR for nucleotide datasets and JTT for amino acid datasets.

Table S2.1: **Comparison of UPP variants on representative full-length datasets with respect to alignment SP-error, tree error, and TC scores.** All criteria (errors and scores) given as percentages. The default setting for UPP is denoted UPP(Default); it uses a backbone of size 1000, uses PASTA to compute the backbone alignment, and the ensemble of HMMs technique. UPP(Fast) is obtained by using backbones of size 100 and keeping all other settings constant. The "NoDecomp" versions of these two methods replace the ensemble of HMMs technique with a single HMM. The "Disjoint" versions of these methods use HMMs computed on disjoint subset alignments of at most ten sequences. UPP(Default,Clade) uses clade-based decompositions to generate the sequence subsets. Maximum likelihood trees are estimated using RAxML (on the 10 AA datasets) or FastTree (all other datasets) except for HomFam, where we do not compute trees as the reference trees are too poorly supported for these to be meaningful.

| Model condition | Method | Alignment SP-error | ΔFN | TC score |
|---|---|---|---|---|
| 10 AA | UPP(Default) | 24.2 | 3.4 | 11.4 |
| 10 AA | UPP(Default,Clade) | 24.2 | X | 11.9 |
| 10 AA | UPP(Default,No Decomp) | 24.5 | 5.2 | 11.0 |
| 10 AA | UPP(Default,Disjoint) | 24.3 | X | 11.6 |
| ROSE AA | UPP(Default) | 2.9 | 1.8 | 2.6 |
| ROSE AA | UPP(Default,Clade) | 2.9 | 1.5 | 2.6 |
| ROSE AA | UPP(Default,No Decomp) | 2.8 | 1.4 | 2.5 |
| ROSE AA | UPP(Default,Disjoint) | 3.2 | 1.9 | 2.2 |
| CRW | UPP(Default) | 12.5 | 7.8 | 1.4 |
| CRW | UPP(Default,Clade) | 12.5 | 8.0 | 1.2 |
| CRW | UPP(Default,No Decomp) | 13.3 | 16.5 | 0.9 |
| CRW | UPP(Default,Disjoint) | 12.5 | 7.1 | 1.3 |
| HomFam(19) | UPP(Default) | 23.0 | NA | 46.6 |
| HomFam(19) | UPP(Default,No Decomp) | 25.4 | NA | 44.5 |
| HomFam(19) | UPP(Default,Disjoint) | 21.2 | NA | 49.4 |
| Indel. 10000M2 | UPP(Default) | 3.5 | 0.6 | 1.2 |
| Indel. 10000M2 | UPP(Default,Clade) | 3.5 | 0.5 | 1.2 |
| Indel. 10000M2 | UPP(Default,No Decomp) | 3.3 | 0.5 | 1.4 |
| Indel. 10000M2 | UPP(Default,Disjoint) | 28.2 | 19.9 | 0.3 |
| Indel. 10000M3 | UPP(Default) | 1.3 | 0.2 | 4.6 |
| Indel. 10000M3 | UPP(Default,Clade) | 1.3 | 0.2 | 4.9 |
| Indel. 10000M3 | UPP(Default,No Decomp) | 1.3 | 0.1 | 4.8 |
| Indel. 10000M3 | UPP(Default,Disjoint) | 6.5 | 1.5 | 1.5 |
| Indel. 10000M4 | UPP(Default) | 0.3 | <0.0 | 27.4 |
| Indel. 10000M4 | UPP(Default,Clade) | 0.3 | <0.0 | 27.4 |
| Indel. 10000M4 | UPP(Default,No Decomp) | 0.5 | <0.0 | 30.5 |
| Indel. 10000M4 | UPP(Default,Disjoint) | 0.6 | 0.1 | 11.9 |
| RNASim 10K | UPP(Default) | 9.5 | 0.8 | 0.5 |
| RNASim 10K | UPP(Fast) | 13.3 | 1.2 | 0.2 |
| RNASim 10K | UPP(Default,Clade) | 9.5 | 0.7 | 0.4 |
| RNASim 10K | UPP(Default,No Decomp) | 11.2 | 3.0 | 0.3 |
| RNASim 10K | UPP(Default,Disjoint) | 10.5 | 0.4 | 0.9 |

Table S2.2: **Results for UPP variants on the RNASim datasets.** We show results for different variants of UPP on the RNASim datasets with 10,000 to 1,000,000 sequences. See text for explanation of names of methods and computational platforms used.

| Number seq. | Method | Align. SP-error | FN | $\Delta FN$ | Time (hrs) |
|---|---|---|---|---|---|
| 10,000 | UPP(Fast,NoDecomp) | 13.1% | 14.2% | 3.6% | 0.1 |
| 10,000 | UPP(Default,NoDecomp) | 11.2% | 13.6% | 3.0% | 0.2 |
| 10,000 | UPP(Fast) | 13.3% | 11.8% | 1.2% | 0.9 |
| 10,000 | UPP(Default) | 9.5% | 11.3% | 0.8% | 6.7 |
| 10,000 | UPP(Default,Disjoint) | 10.5% | 11.0% | 0.4% | 3.2 |
| 10,000 | UPP(Default,Clade) | 9.5% | 11.3% | 0.7% | 10.9 |
| 10,000 | UPP(Default,NoEntropy) | 9.5% | 11.1% | 0.6% | 6.3 |
| 10,000 | UPP(Default,NoReweight) | 9.5% | 11.3% | 0.8% | 6.6 |
| 10,000 | UPP(Fast, Mafft-Profile(add)) | 26.2% | 18.0% | 7.4% | 0.2 |
| 10,000 | UPP(Default,Mafft-Profile(add)) | 14.0% | 14.8% | 4.2% | 0.3 |
| 10,000 | UPP(Fast, Mafft-Profile(addfragments)) | 17.8% | 15.5% | 4.9% | 1.0 |
| 10,000 | UPP(Default, Mafft-Profile(addfragments)) | 12.7% | 12.3% | 1.7% | 6.5 |
| 50,000 | UPP(Fast,NoDecomp) | 12.2% | 10.7% | 2.6% | 0.4 |
| 50,000 | UPP(Default,NoDecomp) | 12.0% | 10.5% | 2.5% | 0.9 |
| 50,000 | UPP(Fast) | 12.7% | 9.4% | 1.3% | 4.2 |
| 50,000 | UPP(Default) | 11.2% | 8.6% | 0.5% | 44.0 |
| 50,000 | UPP(Default,Disjoint) | 11.4% | 8.5% | 0.4% | 18.0 |
| 50,000 | UPP(Fast, Mafft-Profile(add)) | 33.6% | 13.8% | 5.7% | 2.1 |
| 50,000 | UPP(Default, Mafft-Profile(add)) | 16.0% | 10.1% | 2.2% | 3.5 |
| 100,000 | UPP(Fast,NoDecomp) | 13.5% | 9.9% | 3.3% | 0.8 |
| 100,000 | UPP(Default,NoDecomp) | 11.2% | 9.4% | 2.8% | 1.9 |
| 100,000 | UPP(Fast) | 13.0% | 8.3% | 1.4% | 8.5 |
| 100,000 | UPP(Default) | 11.1% | 7.6% | 0.7% | 82.3 |
| 100,000 | UPP(Fast,Mafft-Profile(add)) | 40.2% | 10.2% | 3.3% | 10.7 |
| 200,000 | UPP(Fast,NoDecomp) | 12.4% | 8.5% | 2.4% | 1.9 |
| 200,000 | UPP(Default,NoDecomp) | 11.3% | 8.6% | 2.4% | 6.1 |
| 200,000 | UPP(Fast) | 12.5% | 7.6% | 1.4% | 17.9 |
| 200,000 | UPP(Default) | 10.6% | 6.8% | 0.7% | 151.1 |
| 1,000,000 | UPP(Fast,NoDecomp) | 13.0% | 8.4% | 2.8% | *51.6 |
| 1,000,000 | UPP(Default,NoDecomp) | 11.1% | 7.7% | 2.1% | *64.7 |
| 1,000,000 | UPP(Fast) | 12.8% | 7.5% | 2.0% | *286.4 |
| 1,000,000 | UPP(Fast,Disjoint) | 13.1% | 7.2% | 1.6% | *177.7 |
| 1,000,000 | True alignment | 0.0% | 5.6% | 0.0% | 0.0 |

## S2.2 Backbone size

We examined the impact of the backbone size on alignment and tree accuracy on the RNASim dataset (Table S2.2). UPP run in default mode uses a backbone of size 1000, and run in fast mode uses a backbone of size 100. The comparison between UPP(Default) and UPP(Fast) shows that using the larger backbone generally resulted in lower alignment SP-error and tree error than using a smaller backbone. However, the larger backbone increased the running time, often substantially.

## S2.3 Backbone alignment method

We ran UPP(Fast) on the backbone alignments estimated using Clustal-Omega, MAFFT-L-INS-i, MUSCLE, and PASTA on backbone sets of size 100 on the RNASim 10K dataset (Fig. S2.3). We found that UPP using PASTA and MUSCLE backbones resulted in the most accurate UPP alignments, followed very closely by UPP on the MAFFT-L-INS-i backbone. UPP on using the Clustal-Omega backbone, on the other hand, resulted in a distinctively worse alignment. While UPP on PASTA and MUSCLE backbones resulted in the best alignments, UPP on PASTA and MAFFT-L-INS alignments resulted in the best trees. UPP on MUSCLE was close behind, and as before, UPP on Clustal-Omega was distinctly worse. Thus, using PASTA backbones gave the best overall results compared to the other alignment methods.

## S2.4 Backbone and final alignment SP-error

We examined the alignment SP-error of the initial backbone alignment and the resulting alignment SP-error of the final UPP alignment (Fig. S2.4). We found that the backbone alignment SP-error was statistically significantly correlated to the final UPP alignment SP-error (Pearson's correlation coefficient 0.951; p-value of 5.968e-07).

With the exception of the CRW 16S.T dataset, the final alignment SP-error very closely

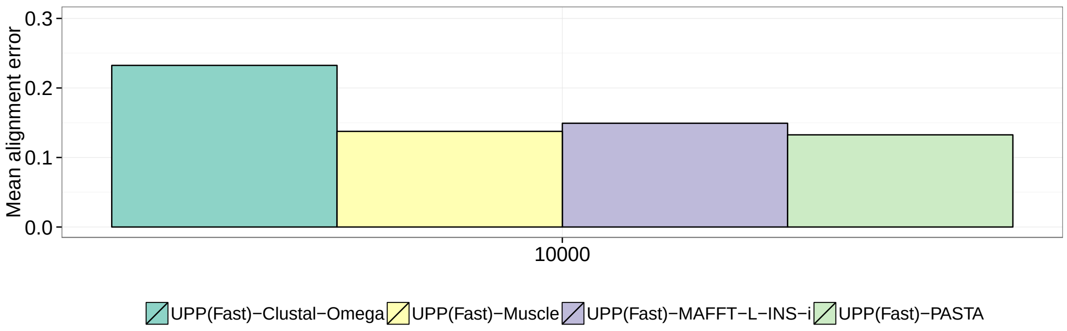


(a) Alignment SP-error

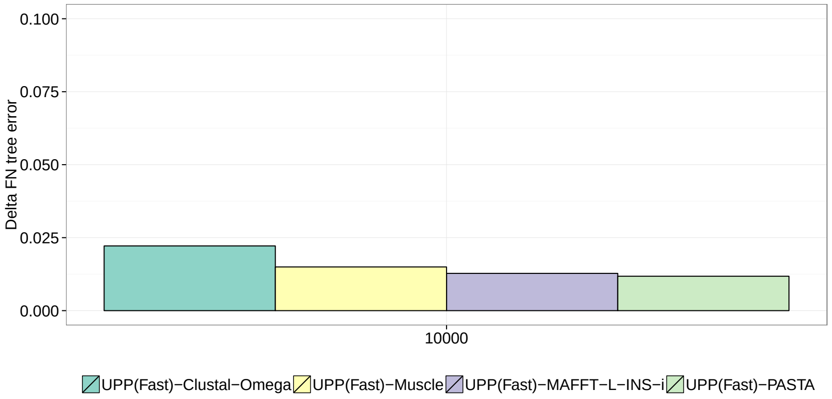


(b) ΔFN tree error

Figure S2.3: **Average alignment SP-error and ΔFN tree error rates of UPP using different alignment methods to estimate the backbone alignment on the RNASim 10K dataset.** All backbones are of size 100.

matches the initial error in the backbone alignment. The explanation is that several clades are omitted from the backbone set due to UPP's restriction of the backbone to sequences considered full-length, so that UPP has difficulty aligning sequences from those clades.

## S2.5 Query sequence alignment method

We compared three different techniques for aligning the query sequences to the backbone alignment within the UPP pipeline: using the ensemble of HMMs technique, using MAFFT-Profile "--add", and using MAFFT-Profile "--addfragments". Table S2.2 and Figure S2.5 showed that the ensemble of HMMs technique resulted in lower alignment SP-error and tree error than MAFFT-Profile, whether using --add or --addfragments. In addition, UPP using the ensemble of HMMs technique made it possible to align 200,000 sequences within 24 hours, but UPP using MAFFT-Profile "--add" was unable to align the 200K dataset in that timeframe, and UPP using MAFFT-Profile "--addfragments" could only align up to 10,000 sequences (Table S2.2). Comparing MAFFT-Profile "--add" and MAFFT-Profile "--addfragments", we found that MAFFT-Profile "--addfragments" resulted in lower alignment SP-error and tree error than MAFFT-add (Fig. S2.5), at a large increase in running time (Table S2.2).

## S2.6 Comparison of clade-based versus centroid edge decomposition.

The centroid edge decomposition does not guarantee that the resulting sequence subsets form monophyletic clades in the backbone tree. Thus, the subset alignments can be polyphyletic or paraphyletic, especially if the backbone tree is unbalanced.

In this section, we compared a centroid edge decomposition to a clade-based decomposition. The clade-based decomposition partitions the backbone tree into two subtrees by breaking the tree on the root node. This process recursively repeats on any subtree that is larger than the maximum alignment subset size (set to 10 for this example).

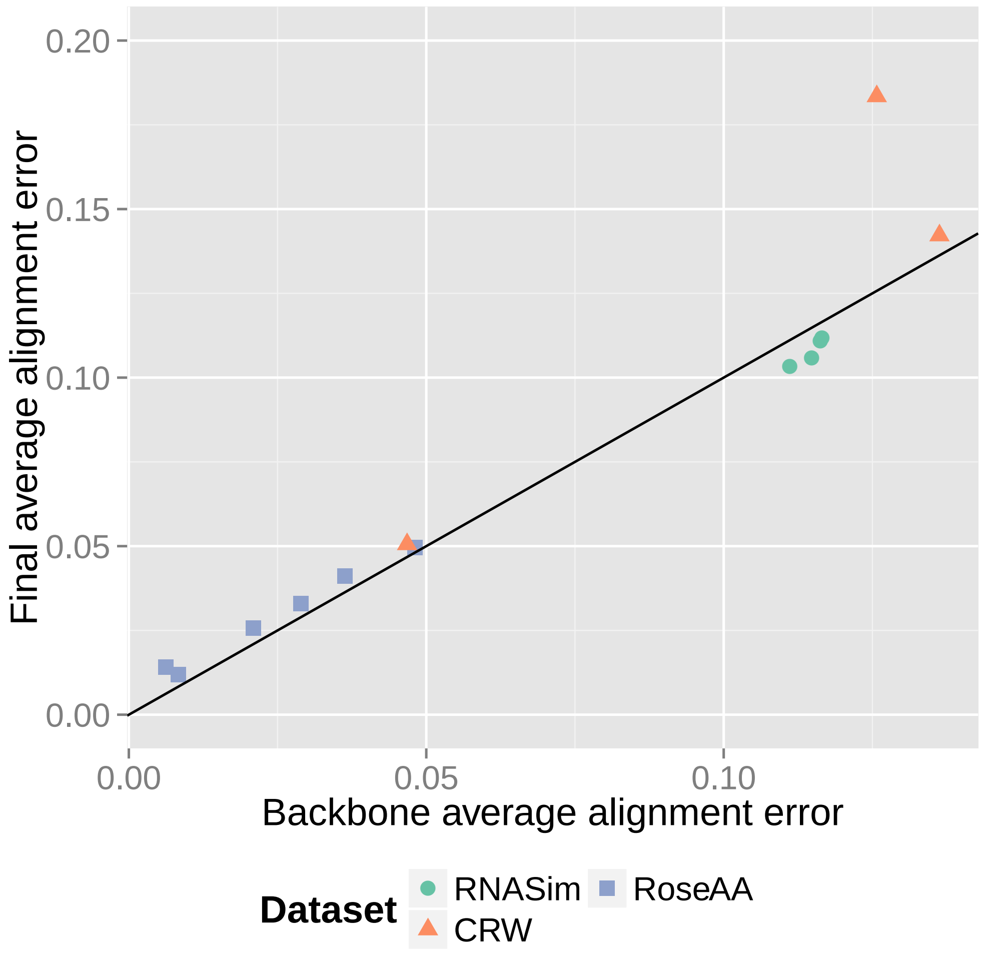


Figure S2.4: **Comparison of initial backbone alignment SP-error and final UPP(Default) alignment SP-error, using PASTA backbones of size 1000.** Each point represents the alignment SP-error for a specific method on a specific dataset. Points below the line represent alignment methods that have a lower alignment SP-error relative to the backbone alignment. Points above the line represent alignment methods that have a higher alignment SP-error relative to the backbone alignment. The majority of the final alignment SP-errors closely tracks the initial backbone alignment SP-errors. The lone exception is on the CRW 16S.T dataset. The Pearson's correlation coefficient for the backbone alignment SP-error versus the final alignment SP-error for the entire collection of points is 0.951 and is statistically significantly correlated (p-value of 5.968e-07; Pearson's product-moment correlation test).

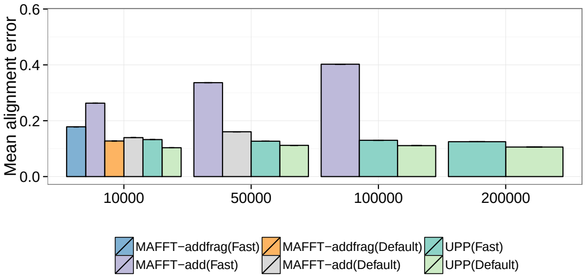


(a) Alignment SP-error

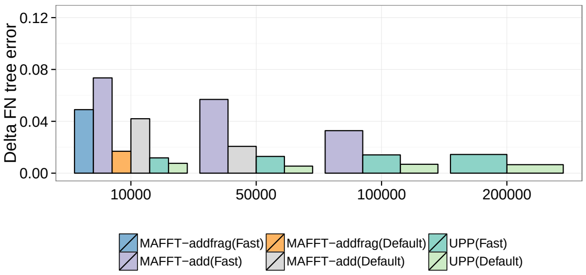


(b) ΔFN tree error

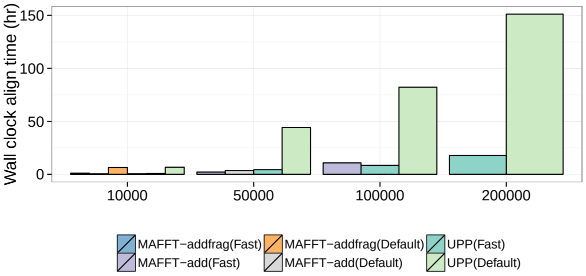


(c) Wall clock running time

Figure S2.5: **Impact of backbone size and query sequence alignment method on alignment SP-error, tree error, and running time.** Methods labeled with "Default" use a backbone size of 1000. Methods labeled with "Fast" use a backbone size of 100. Methods labeled as "MAFFT" used MAFFT as a profile alignment method (either under the "addfrag" or "add" setting) to insert the query sequences into the backbone alignment. ML trees were estimated using FastTree under GTR.

The results on the centroid-edge versus clade-based decomposition for the CRW datasets showed that neither method was consistently better than the other (Fig. S2.6). However, the clade-based decomposition resulted in a larger number of alignment subsets (438 versus 280 for 16S.T), and thus required more time to run (6.1 hours versus 3.9 hours for 16S.T).

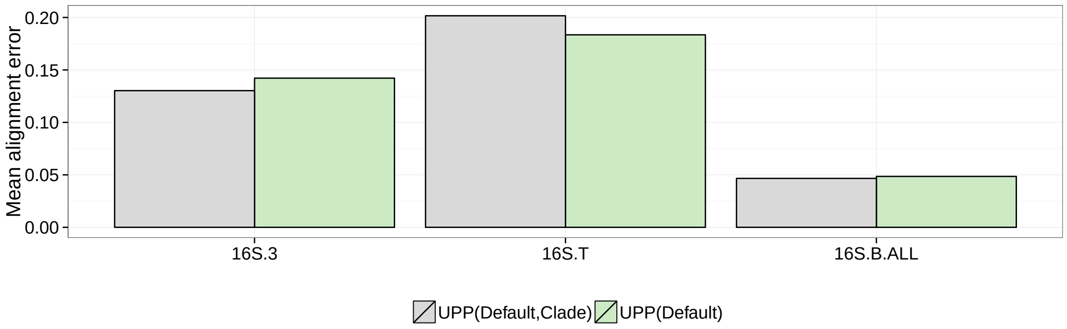


(a) Alignment SP-error

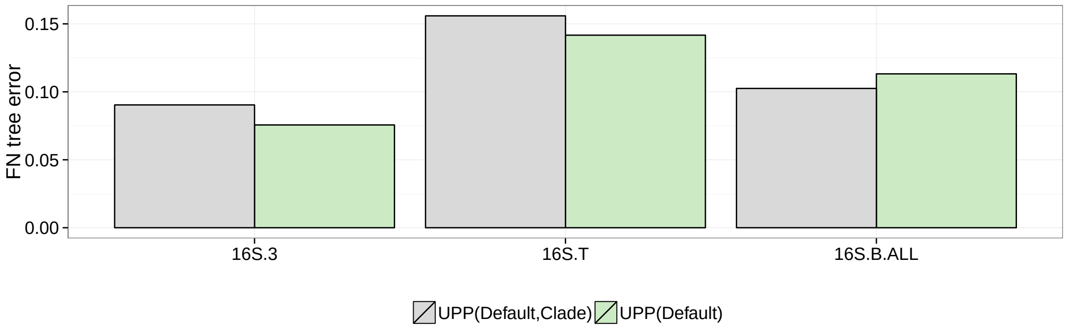


(b) Tree error

Figure S2.6: **Alignment SP-error and tree error for UPP using a centroid-edge decomposition (UPP(Default)) and UPP using clade-based decomposition (UPP(Default,Clade)) on the CRW datasets.** All methods used a backbone size of 1000. ML trees were estimated using FastTree under GTR.

## S2.7 hmmbuild options

We explored two different ways of running hmmbuild. The first way was to disable the relative sequence weight option. By default, hmmbuild downweights similar sequences and upweights

divergent sequences for computing the character frequencies in the profile during the HMM generation. The motivation is to minimize the impact of biased and uneven sampling caused by too many similar sequences being found in the same model. However, this works against the purpose of using an ensemble of HMMs, as the idea is to form profile HMMs from sequence sets have been intentionally partitioned into subsets containing similar sequences. We ran UPP with this flag turned off (labeled “UPP(Default,NoReweight)”).

The second way was related to hmmbuild’s computation of the number of effective sequences used to generate the HMM (called “entropy-weighting”). HMMER, by default, attempts to reach a per site entropy setting of 0.6 bits per consensus position. By default, HMM computes an effective number of sequence that is typically smaller than $N$, the number of sequences in the subset used to generate the HMM model, which has the effect of reducing the HMM score per match. This causes longer alignments to require more hits to receive good scores, but as a side effect this causes short sequences to receive lower scores than expected. We ran UPP with this flag turned off (labeled “UPP(Default,NoEntropy)”).

Figure S2.7 shows the results of the hmmbuild variants on the CRW datasets, and Table S2.2 shows results on the RNASim 10K datasets. There were no differences between methods on the RNASim 10K datasets or the CRW 16S.B.ALL dataset. On the 16S.3 and 16S.T datasets, using the UPP default setting for hmmbuild produced the most accurate trees. Using the no entropy or no reweight setting increased alignment SP-error on the 16S.T dataset and slightly decreased alignment SP-error on the 16S.3 dataset. Thus, changing how hmmbuild was used did not reliably improve the performance of UPP with respect to alignment SP-error or tree error.

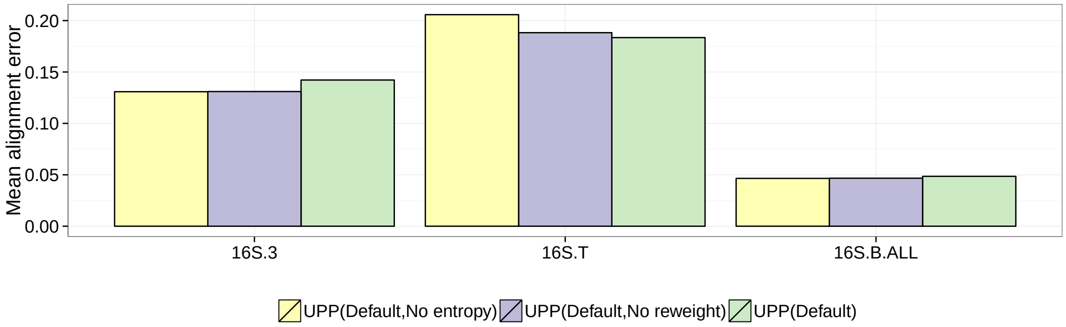


(a) Alignment SP-error

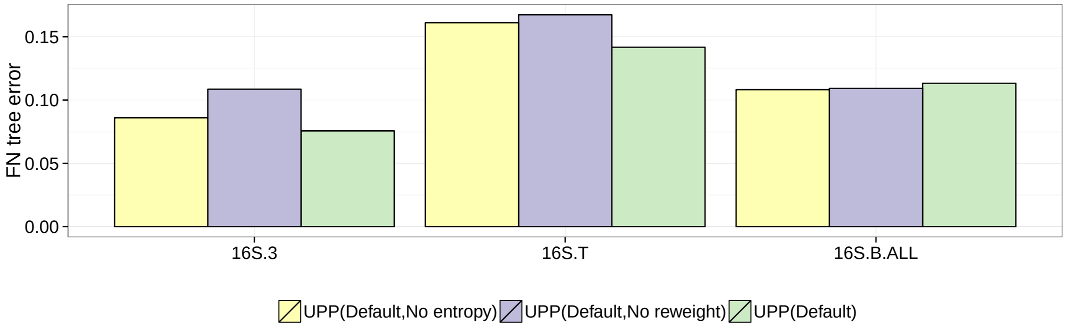


(b) Tree error

Figure S2.7: **Alignment SP-error and tree error for different options of hmmbuild within UPP on the CRW datasets.** UPP(Default,NoReweight) is UPP run with the sequence reweighting flag turned off. UPP(Default,NoEntropy) is UPP run with the entropy-weighting turned off. All methods used a backbone size of 1000. ML trees were estimated using FastTree under GTR.

## S3 PASTA on the 10 AA datasets

UPP's alignment accuracy depends on the accuracy of the backbone alignment. PASTA is an improvement on SATé-II, and both have been studied extensively on nucleotide datasets (*1*); however, there has been less exploration of PASTA on AA datasets, and no studies of SATé-I or SATé-II on AA datasets.

We explored PASTA variants, varying the technique used to estimate alignments on subsets and then to merge alignments together, using the 10 AA datasets with full reference alignments. Initial analyses revealed that MAFFT-L-INS-i gave the best results for producing the subset alignments. We then evaluated techniques for merging alignments, including Opal (*2*), MUSCLE (*3*), or COBALT (*4*).

We ran PASTA under default settings (no starting tree, subset size 200, MAFFT-L-INS-i to align subsets, FastTree to compute trees in each iteration, and running for three iterations), varying only the alignment merger technique. The software version numbers and commands used within PASTA to align the sequences and merge the subsets are given in Section S3. ML trees were estimated on the alignments using RAxML under JTT, LG, or WAG models of protein evolution (using ProtEST (*5*) to select the amino acid substitution model).

We found that while all PASTA variants resulted in alignments with comparable accuracy, RAxML maximum likelihood trees on PASTA using MUSCLE to merge subalignments resulted in the most accurate trees (Fig. S3.1). We refer to this version as "PASTA-MUSCLE."

We then compared PASTA-MUSCLE to alignments and trees computed using standard MSA methods followed by RAxML for maximum likelihood. PASTA-MUSCLE and MAFFT-L-INS-i gave the most accurate alignments, but PASTA-MUSCLE resulted in the most accurate trees (Fig. S3.2). Thus, we used PASTA-MUSCLE for amino acid sequence datasets.

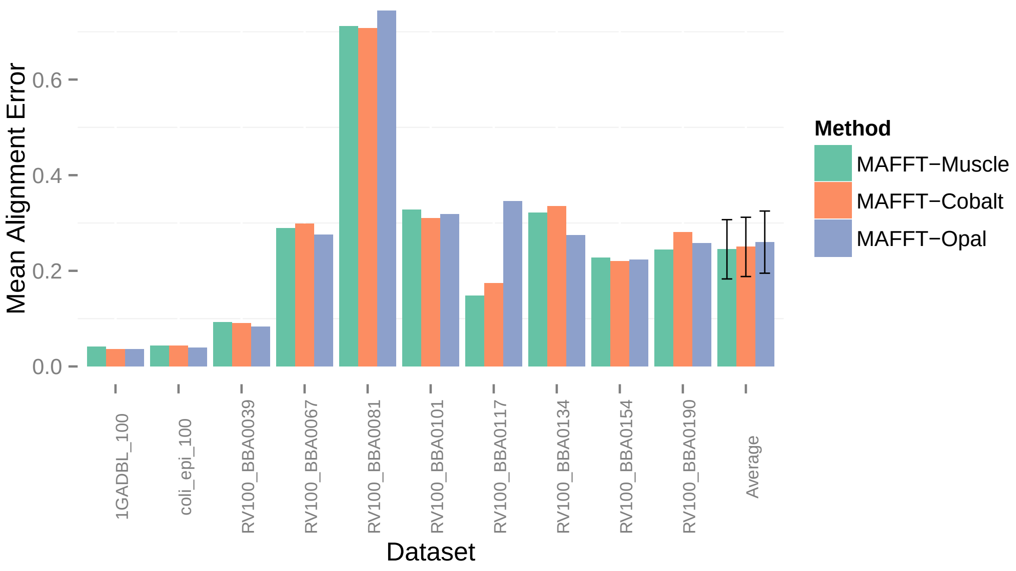


(a) Mean alignment error of PASTA variants

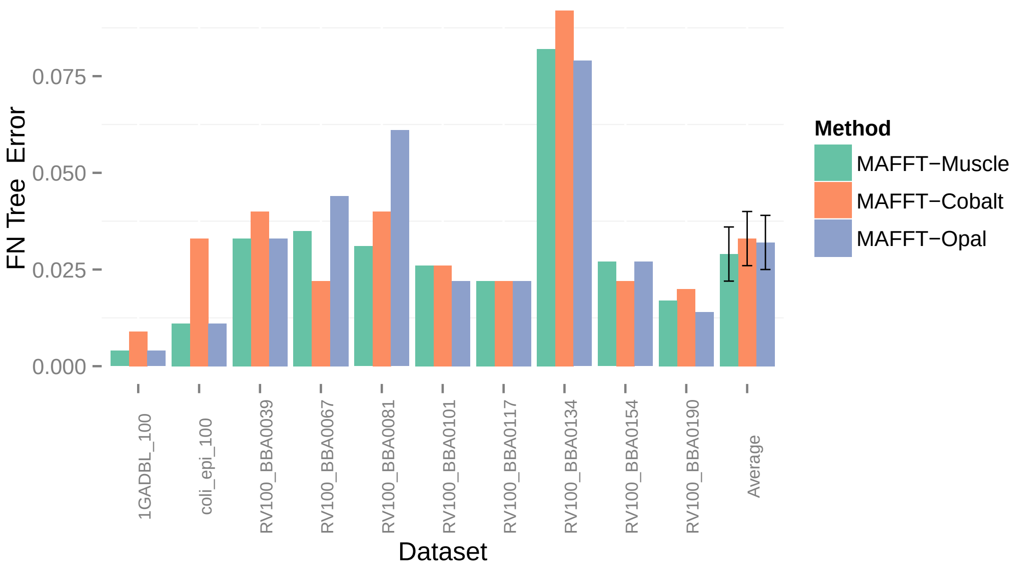


(b) Tree FN error of PASTA variants

Figure S3.1: **Alignment SP-error and tree error for PASTA variants on the 10 AA datasets with full reference alignments.** We show the results for PASTA using MAFFT to align the alignment subsets, and then using either Muscle (labeled as "MAFFT-Muscle"), Cobalt (labeled as "MAFFT-Cobalt"), or Opal (labeled as "MAFFT-Opal") to merge the subalignments. ML trees were estimated using RAxML under amino acid substitution models selected using ProtEST.

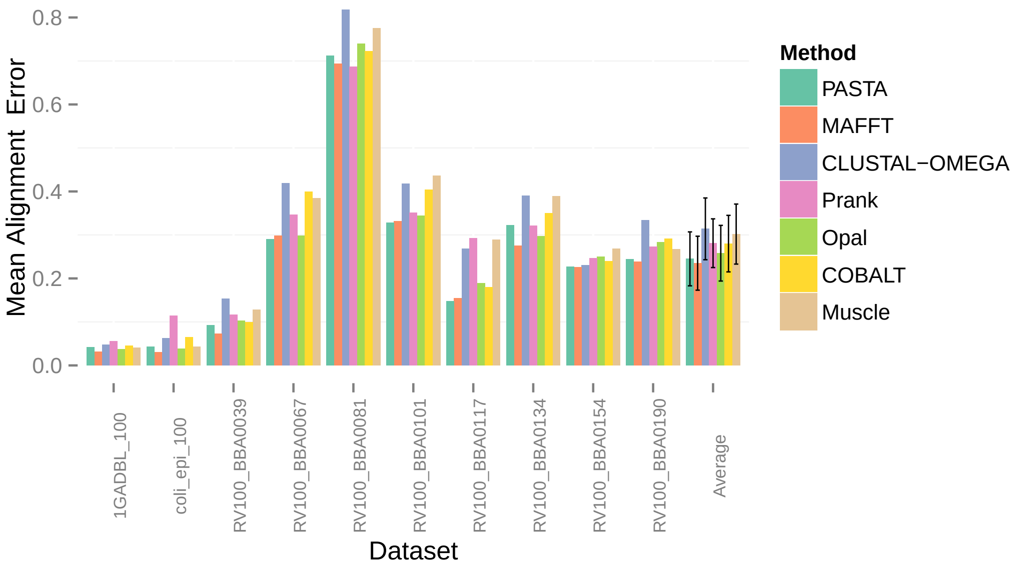


(a) Mean alignment error

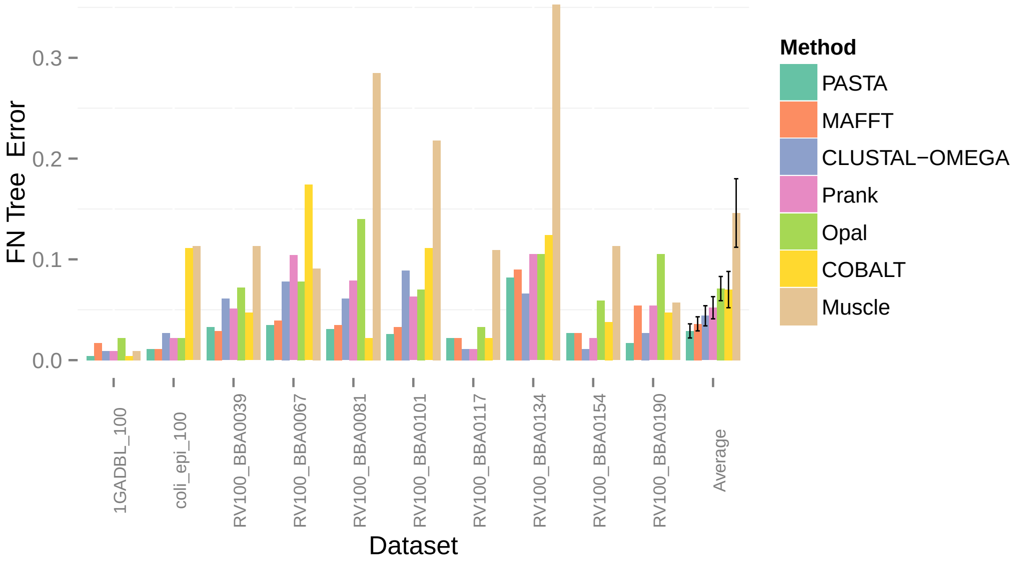


(b) Tree FN error

Figure S3.2: **Alignment SP-error and tree error of different methods on the 10 AA datasets with full reference alignments.** PASTA used MAFFT-L-INS-i to align subalignments, and MUSCLE to merge subalignments. ML Trees were estimated using RAxML under amino acid substitution models selected using ProtEST.

**PASTA commands.** We present information on the external software we used in running PASTA. Each dataset was aligned (when possible) using Opal (*2*) version 2.0.0, Clustal-Omega (*6*) version 1.0.2, MAFFT (*7–9*) version 6.857b, Cobalt (*4*) version 2.0.1, MUSCLE (*3, 10*) version 3.8.31, PRANK (*11*) version 100802 and PASTA version 1.0 (*1*). Due to a bug in earlier versions of MAFFT 6.956b, MAFFT-Profile and MAFFT-default were run using MAFFT version 7.143.

The commands used for the experiments in this section are given below.

- **Clustal-Omega**: *clustalo -align -i<input_sequence> -o <output_alignment>*
- **MAFFT**: *mafft --localpair --maxiterate 1000 --ep 0.123 <input_sequence> > <output_alignment>*
- **Opal**: *java -Xmx20g -jar opal.jar --in <input_sequences> --out <output_alignment>*
- **MUSCLE**: *muscle -in <input_sequence> -out <output_alignment>*
- **Cobalt**: *cobalt -i <input_sequence> -rpsdb <cdd_clique_0.75> > output_alignment>*
- **Prank**: *prank -once -noxml -notree -nopost +F -quiet -matinitsize=5 -protein -d=<input_sequence> -o=<output_alignement>*
- **RaxML**: *raxml -m PROTGAMMA<model> -n ml -s <output_phylip> -T2 -w <working_directory>*
- **PASTA**: *python run_pasta.py -o <output_directory> -i <input_sequences> -t <starting_tree> --auto --num-cpus=12 --datatype=<molecule_type>*

# S4 Supplemental Tables

Table S4.1: **The FN tree error rates (as a percentage) for the 10AA and CRW biological datasets.** The reference trees for these datasets were obtained from previous studies, and were computed using RAxML with bootstrapping on the reference alignments and then restricted to the branches with at least 75% bootstrap support.

| Model | Dataset | UPP(Default) | PASTA | MAFFT | Muscle | Clustal-Omega |
|---|---|---|---|---|---|---|
| 10 AA | 1GADBL_100 | 0.0 | 0.9 | 0.9 | 1.7 | 0.9 |
| 10 AA | coli_epi_100 | 1.1 | 1.1 | 0.0 | 0.0 | 0.0 |
| 10 AA | RV100_BBA0039 | 4.7 | 2.9 | 1.1 | 12.3 | 2.2 |
| 10 AA | RV100_BBA0067 | 1.7 | 1.3 | 3.5 | 7.0 | 3.5 |
| 10 AA | RV100_BBA0081 | 7.9 | 9.2 | 4.4 | 28.1 | 6.6 |
| 10 AA | RV100_BBA0101 | 6.3 | 3.3 | 3.0 | 19.6 | 5.5 |
| 10 AA | RV100_BBA0117 | 2.2 | 1.1 | 2.2 | 14.1 | 3.3 |
| 10 AA | RV100_BBA0134 | 6.6 | 9.7 | 3.9 | 32.6 | 9.5 |
| 10 AA | RV100_BBA0154 | 2.2 | 1.1 | 2.2 | 6.5 | 1.6 |
| 10 AA | RV100_BBA0190 | 1.4 | 2.0 | 2.4 | 4.4 | 2.4 |
| CRW | 16S.3 | 7.6 | 7.7 | 8.1 | 8.2 | 32.0 |
| CRW | 16S.B.ALL | 11.3 | 9.9 | 24.1 | 10.2 | 49.7 |
| CRW | 16S.T | 14.2 | 7.5 | 7.8 | 8.0 | 30.2 |

Table S4.2: **The alignment SP-error for the full-length biological datasets.** We report the alignment error on the full-length biological datasets. Muscle failed to align two of the HomFam datasets; we denote this with an "X".

| Model | Dataset | UPP(Default) | PASTA | MAFFT | Muscle | Clustal-Omega |
|---|---|---|---|---|---|---|
| 10 AA | 1GADBL_100 | 4.6 | 2.9 | 3.1 | 4.1 | 4.7 |
| 10 AA | coli_epi_100 | 4.0 | 4.1 | 3.1 | 4.3 | 5.0 |
| 10 AA | RV100_BBA0039 | 8.6 | 7.7 | 7.2 | 12.8 | 8.4 |
| 10 AA | RV100_BBA0067 | 27.3 | 26.5 | 29.3 | 38.4 | 29.4 |
| 10 AA | RV100_BBA0081 | 71.5 | 73.8 | 69.6 | 77.6 | 68.9 |
| 10 AA | RV100_BBA0101 | 32.0 | 29.7 | 33.3 | 43.6 | 33.0 |
| 10 AA | RV100_BBA0117 | 16.4 | 18.7 | 17.0 | 28.9 | 19.4 |
| 10 AA | RV100_BBA0134 | 30.4 | 27.4 | 26.4 | 38.9 | 30.2 |
| 10 AA | RV100_BBA0154 | 23.6 | 23.7 | 22.9 | 26.9 | 20.5 |
| 10 AA | RV100_BBA0190 | 23.8 | 25.7 | 23.5 | 26.7 | 23.9 |
| CRW | 16S.3 | 14.2 | 13.2 | 25.2 | 25.8 | 43.2 |
| CRW | 16S.B.ALL | 4.9 | 5.3 | 29.4 | 33.7 | 39.9 |
| CRW | 16S.T | 18.3 | 19.9 | 30.4 | 32.8 | 46.9 |
| HomFam | aat | 22.8 | 25.7 | 20.4 | 71.2 | 24.3 |
| HomFam | Acetyltransf | 46.4 | 37.3 | 62.8 | 71.9 | 38.8 |
| HomFam | adh | 35.6 | 9.1 | 1.2 | 19.0 | 1.4 |
| HomFam | aldosered | 9.8 | 11.2 | 11.0 | 39.1 | 16.1 |
| HomFam | biotin_lipoyl | 7.9 | 8.0 | 8.1 | 8.6 | 6.8 |
| HomFam | blmb | 33.1 | 37.4 | 36.4 | 70.8 | 48.9 |
| HomFam | ghf13 | 41.7 | 35.2 | 35.8 | 69.6 | 36.0 |
| HomFam | gluts | 8.8 | 18.3 | 15.0 | 48.5 | 6.8 |
| HomFam | hla | 0.0 | 0.0 | 0.0 | 5.0 | 0.0 |
| HomFam | hom | 5.3 | 3.3 | 3.6 | 8.0 | 10.7 |
| HomFam | myb_DNA-binding | 5.4 | 8.5 | 13.1 | 17.4 | 17.4 |
| HomFam | p450 | 25.6 | 42.9 | 44.1 | 92.5 | 44.9 |
| HomFam | PDZ | 16.7 | 21.4 | 23.2 | 49.7 | 25.0 |
| HomFam | Rhodanese | 36.2 | 31.5 | 34.6 | 58.8 | 42.8 |
| HomFam | rrm | 21.0 | 23.0 | 24.4 | 59.3 | 47.5 |
| HomFam | rvp | 27.7 | 20.4 | 28.8 | X | 26.8 |
| HomFam | sdr | 27.8 | 27.1 | 45.7 | 76.8 | 49.3 |
| HomFam | tRNA-synt_2b | 51.9 | 42.1 | 50.2 | 51.1 | 53.9 |
| HomFam | zf-CCHH | 13.8 | 14.2 | 12.6 | X | 32.0 |

Table S4.3: **The TC score for the full-length biological datasets.** We report the TC score (the percentage of the sites in the reference alignment that appear in the estimated alignment) on the full-length biological datasets. Muscle failed to align two of the HomFam datasets; we denote this with an "X".

| Model | Dataset | UPP(Default) | PASTA | MAFFT | Muscle | Clustal-Omega |
|---|---|---|---|---|---|---|
| 10 AA | 1GADBL_100 | 27.8 | 29.0 | 30.6 | 32.2 | 31.6 |
| 10 AA | coli_epi_100 | 46.0 | 47.3 | 46.7 | 48.0 | 44.0 |
| 10 AA | RV100_BBA0039 | 0.0 | 1.7 | 2.8 | 0.0 | 0.7 |
| 10 AA | RV100_BBA0067 | 5.9 | 7.0 | 4.2 | 0.7 | 6.0 |
| 10 AA | RV100_BBA0081 | 0.5 | 1.1 | 1.9 | 0.5 | 0.8 |
| 10 AA | RV100_BBA0101 | 1.7 | 0.1 | 0.1 | 0.0 | 1.5 |
| 10 AA | RV100_BBA0117 | 0.0 | 0.0 | 0.0 | 0.0 | 0.0 |
| 10 AA | RV100_BBA0134 | 0.1 | 1.0 | 0.1 | 0.0 | 1.0 |
| 10 AA | RV100_BBA0154 | 12.1 | 12.7 | 12.9 | 10.6 | 11.9 |
| 10 AA | RV100_BBA0190 | 7.9 | 9.6 | 10.3 | 3.7 | 8.3 |
| CRW | 16S.3 | 1.0 | 3.4 | 0.9 | 0.4 | 0.1 |
| CRW | 16S.B.ALL | 1.4 | 1.4 | 0.2 | 1.2 | 0.0 |
| CRW | 16S.T | 1.0 | 1.0 | 0.7 | 0.2 | 0.0 |
| HomFam | aat | 39.9 | 34.0 | 38.9 | 1.1 | 26.3 |
| HomFam | Acetyltransf | 18.3 | 21.8 | 8.7 | 2.6 | 14.8 |
| HomFam | adh | 34.7 | 84.0 | 97.3 | 66.7 | 96.5 |
| HomFam | aldosered | 58.5 | 47.9 | 51.0 | 13.5 | 26.9 |
| HomFam | biotin_lipoyl | 51.8 | 52.7 | 53.6 | 52.7 | 57.1 |
| HomFam | blmb | 27.3 | 22.1 | 23.8 | 5.2 | 9.0 |
| HomFam | ghf13 | 16.0 | 31.3 | 24.9 | 7.5 | 20.6 |
| HomFam | gluts | 72.8 | 42.6 | 56.2 | 13.6 | 75.3 |
| HomFam | hla | 100.0 | 100.0 | 100.0 | 86.0 | 100.0 |
| HomFam | hom | 48.0 | 53.1 | 53.1 | 50.0 | 40.8 |
| HomFam | myb_DNA-binding | 72.1 | 65.6 | 57.4 | 52.5 | 41.0 |
| HomFam | p450 | 35.7 | 0.4 | 10.9 | 0.0 | 10.4 |
| HomFam | PDZ | 53.6 | 45.5 | 46.4 | 14.5 | 27.3 |
| HomFam | Rhodanese | 36.6 | 41.2 | 30.6 | 22.7 | 22.7 |
| HomFam | rrm | 11.5 | 10.8 | 9.6 | 1.3 | 3.2 |
| HomFam | rvp | 31.8 | 40.9 | 33.3 | X | 40.2 |
| HomFam | sdr | 19.7 | 26.0 | 11.1 | 0.6 | 5.5 |
| HomFam | tRNA-synt_2b | 15.2 | 13.7 | 14.3 | 21.0 | 13.3 |
| HomFam | zf-CCHH | 38.5 | 46.2 | 48.7 | X | 12.8 |

# References and Notes


1. S. Mirarab, N. Nguyen, T. Warnow, *Research in Computational Molecular Biology* (Lecture Notes in Computer Science, Springer International Publishing, Switzerland, 2014), vol. 8394, pp. 177–191.

2. T. J. Wheeler, J. D. Kececioglu, *Bioinformatics (Oxford, England)* **23**, i559 (2007).

3. R. Edgar, *BMC Bioinformatics* **5**, 113 (2004).

4. J. S. Papadopoulos, R. Agarwala, *Bioinformatics (Oxford, England)* **23**, 1073 (2007).

5. J. A. Cuff, E. Birney, M. E. Clamp, G. J. Barton, *Bioinformatics (Oxford, England)* **16**, 111 (2000).

6. F. Sievers, *et al.*, *Molecular Systems Biology* **7** (2011).

7. K. Katoh, K. Misawa, K.-i. Kuma, T. Miyata, *Nucleic Acids Research* **30**, 3059 (2002).

8. K. Katoh, K.-i. Kuma, H. Toh, T. Miyata, *Nucleic Acids Research* **33**, 511 (2005).

9. K. Katoh, M. C. Frith, *Bioinformatics (Oxford, England)* **28**, 3144 (2012).

10. R. Edgar, *Nucleic Acids Research* **32**, 1792 (2004).

11. A. Löytynoja, N. Goldman, *Proceedings of the National Academy of Sciences of the United States of America* **102**, 10557 (2005).
